# Comparative genomics and proteomics of *Helicobacter mustelae*, an ulcerogenic and carcinogenic gastric pathogen

**DOI:** 10.1186/1471-2164-11-164

**Published:** 2010-03-10

**Authors:** Paul W O'Toole, William J Snelling, Carlos Canchaya, Brian M Forde, Kim R Hardie, Christine Josenhans, Robert LJ Graham, Geoff McMullan, Julian Parkhill, Eugenio Belda, Stephen D Bentley

**Affiliations:** 1 Department of Microbiology, & Alimentary Pharmabiotic Centre, University College Cork, Cork, Ireland; 2 School of Biomedical Sciences, University of Ulster, Coleraine, County Londonderry, BT52 1SA, N. Ireland, UK; 3 Department of Biochemistry, Genetics and Immunology, University of Vigo, 36310 Vigo, Spain; 4 School of Molecular Medical Sciences, University of Nottingham, Centre for Biomolecular Sciences, Nottingham NG7 2RH, UK; 5 Hannover Medical School, Department for Medical Microbiology and Hospital Epidemiology, Hannover, Germany; 6 Pathogen Genomics, Wellcome Trust Sanger Institute, Wellcome Trust Genome Campus, Hinxton, CB10 1SA, UK; 7 Current Address:Cavanilles Institute of Biodiversity and Evolutionary Biology, Department of Genetics, University of Valencia, Valencia, Spain

## Abstract

**Background:**

*Helicobacter mustelae *causes gastritis, ulcers and gastric cancer in ferrets and other mustelids. *H. mustelae *remains the only helicobacter other than *H. pylori *that causes gastric ulceration and cancer in its natural host. To improve understanding of *H. mustelae *pathogenesis, and the ulcerogenic and carcinogenic potential of helicobacters in general, we sequenced the *H. mustelae *genome, and identified 425 expressed proteins in the envelope and cytosolic proteome.

**Results:**

The *H. mustelae *genome lacks orthologs of major *H. pylori *virulence factors including CagA, VacA, BabA, SabA and OipA. However, it encodes ten autotransporter surface proteins, seven of which were detected in the expressed proteome, and which, except for the Hsr protein, are of unknown function. There are 26 putative outer membrane proteins in *H. mustelae*, some of which are most similar to the Hof proteins of *H. pylori*. Although homologs of putative virulence determinants of *H. pylori *(NapA, plasminogen adhesin, collagenase) and *Campylobacter jejuni *(CiaB, Peb4a) are present in the *H. mustelae *genome, it also includes a distinct complement of virulence-related genes including a haemagglutinin/haemolysin protein, and a glycosyl transferase for producing blood group A/B on its lipopolysaccharide. The most highly expressed 264 proteins in the cytosolic proteome included many corresponding proteins from *H. pylori*, but the rank profile in *H. mustelae *was distinctive. Of 27 genes shown to be essential for *H. pylori *colonization of the gerbil, all but three had orthologs in *H. mustelae*, identifying a shared set of core proteins for gastric persistence.

**Conclusions:**

The determination of the genome sequence and expressed proteome of the ulcerogenic species *H mustelae *provides a comparative model for *H. pylori *to investigate bacterial gastric carcinogenesis in mammals, and to suggest ways whereby *cag *minus *H. pylori *strains might cause ulceration and cancer.

The genome sequence was deposited in EMBL/GenBank/DDBJ under accession number FN555004.

## Background

The genus *Helicobacter *contains over 50 species, and a large number of candidate or unclassified species, which have been isolated from a wide range of vertebrate hosts (reviewed by Solnick and Vandamme [1]). The type species is *Helicobacter pylori *(reviewed in ref. [2]), which is a causative agent for duodenal ulcers & peptic ulcers [3,4], is a risk factor for gastric adenocarcinoma [5] and for B cell MALT lymphoma [6]. The majority of *Helicobacter *species are not found in the stomach [7]. One enterohepatic species, *H. hepaticus *has been shown to cause chronic active hepatitis and typhocolitis in infected mice [8], development of liver carcinomas in infected mice [9], and induction of inflammatory bowel disease symptoms in mice [10]. This contrasts with the cases of other extragastric *Helicobacter *species, for example, with the association of *"H. rappini" *(taxonomic name not validly published) with disease in humans and companion animals (reviewed in ref. [7]). All of the gastric *Helicobacter *species produce a potent urease, as an acid protection mechanism, whereas the large number of enteric species (gastrointestinal, intestinal, hepatic and biliary) are not uniformly urease positive [7].

The ferret (*Mustela putorius*) is a valuable element in comparative medicine, providing *inter alia *models for human influenza [11] and infectious gastritis [12,13]. *H. mustelae *is a gastric pathogen of ferrets, and was the second member of the genus identified [14-16]. *H. mustelae *cells are smaller and typically less helical than those of *H. pylori*, with lateral as well as bipolar flagella [17]. *H. mustelae *is virtually endemic in ferrets [12,18] and other mustelids [19], and like *H. pylori*, stimulates a humoral immune response [13], including in naturally infected animals [18], which does not clear the infection. Experimentally infected ferrets develop a gastritis which closely resembles the diffuse antral gastritis seen in some adults, and in children [20]. Ferret gastric epithelial cell proliferation increases upon *H. mustelae *infection [21], and the bacterium has been linked to gastric adenocarcinoma [22] and MALT lymphoma [23] in the infected ferret. Ulcer formation in *H. mustelae*-infected ferrets is also common; *H. mustelae *infection of ferrets is the only natural model of *Helicobacter*-associated ulcer disease, making it a unique model [13]. Although other recently discovered gastric *Helicobacter *species from marine mammals, pigs and companion animals may also cause gastric ulcers and cancer, the knowledge base and tractability of the ferret makes it an attractive animal model for human gastric disease due to *Helicobacter *infection [24].

Despite being isolated from the ferret stomach, phylogenetic analysis of *H. mustelae *based upon the 16S rRNA gene positions it, with *H. suncus*, in a clade within the enteric helicobacters [7,25]. Interestingly, phylogenetic analysis based upon the 23S rRNA gene resulted in a discordant tree structure (from that based upon the 16S rRNA gene; [26]), in which *H. mustelae *was positioned even deeper among the enteric helicobacters. Another interesting outcome of that study was the positioning of another ε-proteobacterium, *Wolinella succinogenes*, between *H. pylori *and *H. hepaticus*, when orthology levels of 870 core proteins were analyzed [26]. *H. mustelae *was not included in that analysis because a genome sequence was not available.

Driven to a large extent by the desire to understand their pathogenesis at a molecular level, many of the ε-proteobacteria have been subjected to genomic analyses. In addition to two early *H. pylori *genome sequence determinations [27,28], draft or complete genome sequences for 11 additional *H. pylori *strains are lodged with NCBI Genome Projects http://www.ncbi.nlm.nih.gov/Entrez/. However no whole genome sequence for gastric *Helicobacter *species other than *H. pylori*, and the closely related *H. acinonychis*, is available so far. Genome sequence projects of an additional five extragastric species (*H. pullorum*, *H. bilis*, *H. winghamensis*, *H, canadensis*, and *H. cinaedi*) are currently underway at the Broad Institute http://www.broadinstitute.org/. The genomes of *H. hepaticus *[29], and *H. acinonychis *[30] have been sequenced, as have those of *Campylobacter jejuni *[31,32], *C. lari*, *C. uppsaliensis *and *C. coli *[33], and *W. succinogenes *[34]. Comparative genomic analysis of four of these *Campylobacterales *species - *H. pylori*, *H. hepaticus*, *C. jejuni*, *W. succinogenes*, has been informative for identifying core proteins and specific adaptations to pathogenicity or commensalism in the respective species [35].

Relatively few *H. mustelae *strains have been characterized in detail at the molecular level. The genome size of *H. mustelae *was estimated by pulsed-field gel electrophoresis to be in the range of 1.685-1.69 Mb for 15 strains examined [36], and the genome was apparently conserved among the strains at this low discrimination level. A number of presumptive virulence factors were identified in *H. mustelae*. A potent urease UreAB is implicated in acid-tolerance and pathology [37-39]. Ferrets may be therapeutically immunized against *H. mustelae *by administration of *H. pylori *urease protein [40]. The *H. mustelae *type strain 12198 was recently shown to produce a second urease enzyme UreAB2 which acts independently of nickel and accessory proteins. It appears to be representative of an adaptation by *Helicobacter *species that colonize the stomachs of carnivores, in which dietary nickel is limiting [41].

*H. mustelae *produces a surface array composed of ring-shaped protein aggregates of the Hsr protein [42], and which is required for persistent infection by *H. mustelae *in the ferret model [43]. We have shown that this surface protein is antigenically variable [44], suggesting it is subject to antibody pressure. Antigenic variation of the Hsr protein is achieved by recombining cassettes encoding epitopes in the passenger region of this autotransporter protein into the expression site [44]. These cassettes were detected in a 15 kb Hsr locus (HSRL), only a third of which is occupied by the expressed *hsr *gene [44], the rest being devoted to sequences encoding alternative antigens. Another major surface antigen is the flagellum, the major components of which have been well studied [45,46]. Type strains of *H. mustelae *have been shown to produce a monofucosyl A type 1 histo-blood group epitope in their LPS [47] and anti-gastric auto-antibodies are elicited by the type strain 12198 (ATCC 43772) [48]. The structure of lipid A of *H. mustelae *strain 43772 differs from that of the lipid A of *H. pylori *[49].

Despite a limited genome-wide mutagenesis approach [50], relatively little about the genomic basis for *H. mustelae *persistence and pathogenesis in the ferret is known, since this organism has not benefitted from the pathogenomics approach that was applied to other *Helicobacter *species [51]. We report here the genome sequence of *H. mustelae *type strain 12198, which is the first whole genome of a non-*H. pylori*-*H. acinonychis *gastric *Helicobacter *species. In addition, we compare the expressed proteome of *H. mustelae *with that of *H. pylori*. These data clarify the species-specific and host-specific adaptations by these gastric helicobacters, consolidate the phylogenomics of the genus, and significantly enhance the value of the ferret model for investigating helicobacter-related gastric disease.

## Results and Discussion

### General *H. mustelae *Genome Features

**Figure 1:**
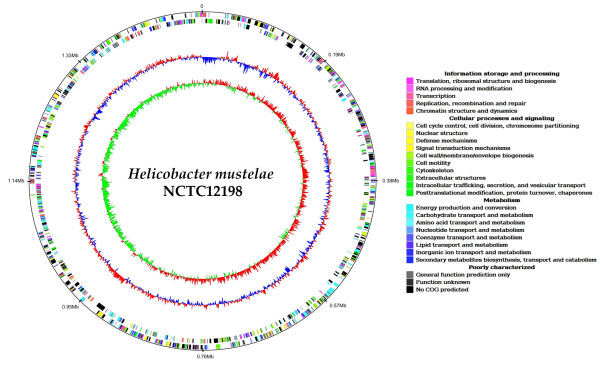
**Circular genome atlas of *H. mustelae***. Rings: 1, Nucleotide co-ordinates in megabase pairs; 2, ORF distribution, plus strand; 3, ORF distribution, negative strand; 4, GC% deviation; 5, GC skew. ORFs are coloured as per colour palette based on COG classifications.

The general features of the *H. mustelae *genome are summarized in Table 1, and compared to selected other genomes of members of the *Campylobacterales*. The *H. mustelae *genome comprises a single circular chromosome of 1,578,097 base-pairs, and like most strains of the ε-proteobacteria selected for sequencing thus far, is plasmid-free. The GC content of the *H. mustelae *genome is the second highest among the *Campylobacterales *analyzed herein, and is among the highest values reported for members of the genus [7]. All the *Campylobacterales *genomes sequenced to date have similarly high coding densities. *H. mustelae *has slightly higher mean predicted CDS length than many other related bacteria, aided by the fact that the *H. mustelae *genome encodes some of the largest proteins ever recorded in this bacterial group (see below). Laterally acquired DNA in bacteria can be identified by local anomalies in GC mol% content, and is often associated with IS elements or tRNA genes [52]. In pathogenic bacteria in general, such islands are typically associated with significant augmentation in virulence capability [53], and the *H. pylori cag *pathogenicity island is a key determinant of increased potential to cause more severe pathology including gastric ulcers and cancer [54]. The *cag *pathogenicity island of *H. pylori *is not present in *H. mustelae*, nor in any of the other helicobacters or campylobacters, including *H. acinonychis *which diverged relatively recently (ca. 200,00 years) from *H. pylori *[30]. Although searching of the *H. mustelae *genome with the Alien Hunter program initially identified 23 candidate regions with anomalous nucleotide content, none of these are large enough to be considered an island or islets (data not shown), and they are not characterized by linkage to phage genes, integrases or tRNA genes. A representative example is the region from HMU08130 to HMU08170 with a GC content of 41.55 mol%, flanked by direct repeat sequences in HMU08130 and HMU0818; this region encodes a presumptive type I restriction-modification system. The region from HMU08300 to HMU08350 has a GC mol% content of 46.69% and harbours several biosynthesis genes that are also found in *H. pylori*, *H. hepaticus*, *W. succinogenes *and *C. jejuni*. Alien Hunter also detected the primary Hsr locus from HMU08520 to HMU08780. Analysis of the GC mol% content around the genome (Fig. 1) shows several smaller stretches of anomalous GC content, similar to localized deviations in *H. pylori*. However, there is no evidence for a pathogenicity island in *H. mustelae*, in contrast to *H. pylori *and *H. hepaticus*. A curious feature of the *H. mustelae *genome is the asymmetric nature of the GC skew pattern ([G-C]/[G+C]; Fig. 1). The pattern of GC skew is normally symmetrical for bacterial circular chromosomes [55]. The lack of symmetry of the *H. mustelae *GC skew may be indicative of recent genome rearrangement, as suggested for *Yersinia pestis *[56]. This is most likely to be a localized deletion, since in contrast to *Y. pestis*, the GC skew pattern of the *H. mustelae *genome does not suggest a transposed genome region. Lack of large-scale synteny between helicobacter genomes (see Comparative Genomics below) complicates identification of such a presumptive deleted region.

**Table 1:** General features of the *H. mustelae *genome, and those of selected *Campylobacterales*

Species	*Helicobacter mustelae*	*Helicobacter pylori*		*Helicobacter acinonychis*	*Helicobacter hepaticus*	*Campylobacter jejuni*	*Wolinella succinogenes*
Strain	ATCC43772^†^ 12198	26695 [28]	J99 [27]	Sheeba [30]	ATCC 51449 [29]	NCTC 11168 [31]	DSM 1740 [34]
Host(s)	Ferrets	Human	Human	Large felines	Rodent	Human & avian	Bovine
Genome size (bp)	1,578,097	1,667,867	1,643,831	1,553,928	1,799,146	1,641,481	2,110,355
GC content (%)	42.47	39.0	39.0	38.2	35.9	30.6	48.5
Predicted ORFs	1,403^†^	1,590 (1,552 [143])	1,495	1,611	1,875	1,654	2,046
Coding area (%)	91.9%	91.0	90.8	89.7	93.0	94.3	93.0
Av.gene length (bp)	995	945	998	865	1,082	948	964
Plasmids	None	None*	None*	One	None	None§	None
Phages and phage-like elements	4 phage genes	None	None	41 phage genes in two prophage	3 phage genes	None	Prophage-tRNAmet
IS elements	None	IS605, IS606	IS605 (partial), IS606	ISHa1675	None	Cj0752 (partial)	ISWsu1302, ISWsu1203
Genomic islands	None	cag PAI	cag PAI	HAcG1 integron	HHGI1	None	WsuGI I and II
Regions of deviating GC content	Autotransporter genes, flagellin modification, surface proteins	Genomic islets & islands, DNA-restriction/modification system, translation machinery	Genomic islets & islands, DNA-restriction/modification system, translation machinery	Hypotheticals and plasmid-related genes	Genomic islets & islands, DNA-restriction/modification system, translation machinery	EPS/LOS synthesis & flagella modification	Genomic islets & islands, DNA-restriction/modification system, translation machinery

^†^Without a pseudogene qualifier

*Plasmids have been identified and/or sequenced in many other strains, reviewed in ref. [35].

The paucity of insertion sequence elements and bacteriophage-related genes in *H. mustelae *(Table 1) suggests that these mechanisms are not significant agents of diversity generation in this species, a process which is driven in *H. pylori *by free recombination [57]. Thus it was surprising to identify a CRISPR locus and three *cas *genes (HMU00230-00250) in the *H. mustelae *genome, as these features have recently been identified as a phage resistance mechanism [58]. CRISPR loci have not been annotated in other helicobacter or campylobacter genomes. HMU00670 encodes another potential phage resistance mechanism, a predicted Abortive Infection (Abi) protein. *H. acinonychis *is unusual in having two complete prophages in its genome (one of which is no longer contiguous, due to genome decay), which has been attributed in part to the presence of only six predicted functional restriction-modification loci compared to eleven in *H. pylori *[30]. The *H. mustelae *genome also encodes 6 predicted restriction-modification systems.

### Virulence-related Genes in *H. mustelae*

Members of the Campylobacterales whose genome has been sequenced to date harbour a variable complement of known or inferred virulence factors genes (reviewed comparatively in ref. [35]). Many of these factors have not actually been studied for biological significance except in *H. pylori*, where the linkage to gastric colonization or pathogenesis for some defined traits is generally very clear [2]. The presence in the *H. mustelae *genome of homologues to genes whose products have been linked to colonization, persistence or pathogenesis in related organisms is summarized in Table 2. As noted above, a *cag *pathogenicity island is not present. Relative to *H. pylori*, the *H. mustelae *genome contains a second (additional) urease operon AB2, that contributes to acid resistance [41]. There is no full-length *H. mustelae *ortholog of the vacuolating cytotoxin VacA of *H. pylori*. Homology searches with the *H. pylori vacA *gene identified 10 predicted autotransporter (AT) genes, organized into three cluster, plus the Hsr locus, and one singleton HMU08270 (Additional File 1). HMU04240 is a VacA homologue that lacks an autotransporter domain. Significantly, when the autotransporter beta-barrel domain is excluded from the analysis, six of the VacA homologues detected using the full length VacA sequence (HMU00600, HMU00620, HMU00630, HMU01180, HMU01190 and HMU08270 show no significant identity between their passenger domains and database entries. HMU06680 and HMU06730 are annotated as glycine-rich autotransporters, located in AT cluster 3. Regions of HMU06680 display 32% identity to HP0922 (a toxin-like outer membrane protein/VacA paralog) and significant residue identity in the passenger domain to an immunodominant antigen in *H. bilis *(accession AAQ14336). In addition, two regions of the HMU06680 protein, residues 100-130 and 1339-1494, show ca. 25% residue identity to passenger-domain regions of VacA, and VacA-like proteins in *H. pylori*. Several older studies have suggested that *H. mustelae *does not produce vacuolating cytotoxic activity [59,60], so the biological significance of these VacA-related proteins is unclear. Of the remaining autotransporters, one is the Hsr variable surface antigen HMU08630, in a genetic configuration similar to strain 4298, flanked by cassettes for alternative epitopes [44]. The predicted autotransporter HMU08270 comprises 4,094 amino acids, has no significant identity to database entries, and an unusual autotransporter domain, which is not at the extreme carboxy-terminus. Finally HMU06740 lacks a predictable signal peptidase cleavage site, and is unusually small for an autotransporter protein. The distinctive wealth of this class of secreted protein in *H. mustelae*, the evidence for their production (see below) and the likelihood of their involvement in host interaction, make this bacterium a potentially productive model for exploring autotransporter evolution and biological function.

**Table 2:** Presence of genes related to colonization, persistence or pathogenesis in the *H. mustelae *genome, compared to *H. pylori*

Trait	*H. pylori *locus	Orthologous *H. mustelae *system	Comments
Cag pathogenicity	cag PAI	absent	Cag PAI linked to severity of pathology and disease
Urease production	Urease operon (ure)	UreAB and UreAB2 loci (HMU03050-030110) and HMU13010-13020)	Contributes to acid resistance
Vacuolating cytotoxin	vacA	absent	Cytotoxicity including T-cell inhibition
Autotransporters	VacA and paralogs	10 genes including Hsr locus (Fig. S1)	Two proteins with low identity to VacA; others - function unknown
Outer membrane adhesins	Hop, Hor, Hof, Hom paralogue families	No orthologs of BabA, SabA, OipA or AlpA/B.	Three OMPs related to Hof family; function unknown; three Hor-Hom homolgues
Invasion antigen	None	CiaB HMU0700	*C. jejuni *homolog enhances cell invasion
Neutrophil activation	NapA	HMU1269	Activates neutrophils & contributes oxidative stress resistance
HpaA	HP0797	absent	Putative adhesin and flagellum sheath lipoprotein
Motility	Fla/Flg/Fli/Flh	Fla/Flg/Fli/Flh	Motility is required for gastric colonization
Collagenase secretion	HP0169	HMU02630	Required for *H. pylori *colonization of Mongolian gerbil
Adhesion/tissue damage	-	HMU00160-HMU00170 Hag/Hly	Putative haemagglutinin/haemolysin
Plasminogen binding	HP0508	HMU02820; HMU09010	Could enhance tissue damage by proteolysis

**Figure 2:**
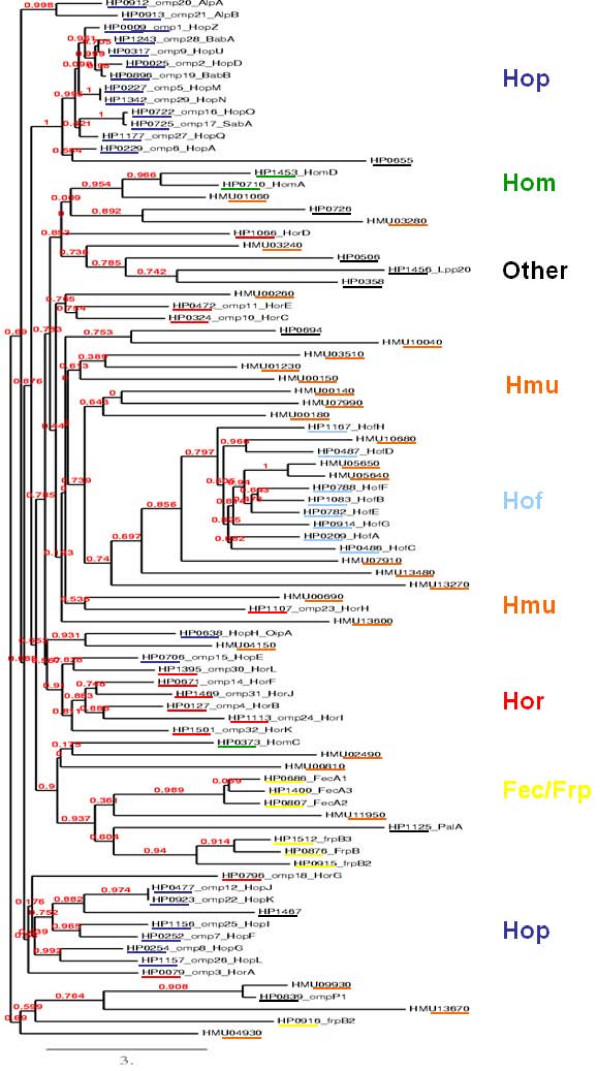
**Phylogeny of *H. mustelae *outer membrane proteins relative to the major OMPs of *H. pylori*, following the classification of Alm et al**. [61]. Protein groups are colour coded, with *H. mustelae *proteins underlined in orange. *H. pylori *OMPs are referred to by 26695 gene number [28]. Phylogeny was rendered by Phyml and TreeDyn at the phylogeny.fr website [128]. The branch length is proportional to the number of substitutions per site

Outer membrane proteins are important for the pathogenesis of *H. pylori*. The genome of *H. mustelae *contains 26 genes that were annotated as encoding putative outer membrane proteins. Phylogenetic analysis of these proteins relative to the categorized *H. pylori *OMPs [61] showed that some of the *H. mustelae *OMPs group with *H. pylori *orthologs (Fig. 2). For example HMU05640, HMU05650, and HMU10680 convincingly cluster in the clade containing the 8 members of the Hof OMP family of *H. pylori*, and the three Hof-related *H. mustelae *proteins show similar size and C-terminal motif to the *H. pylori *Hof proteins (Table 3). Apart from the fact that HP0486 is expressed and is not heat-modifiable [62], suggesting it is not a porin, nothing is known about the function of Hof proteins. Interestingly, a further 12 *H. mustelae *OMPs cluster in two groups either side of the Hof protein clade (Fig. 2). None of the annotated *H. mustelae *OMP sequences position phylogenetically in the tight Hop-containing clade that includes BabA, SabA and OipA, and orthologs of these three adhesins are absent in the *H. mustelae *genome. One Omp in *H. mustelae*, HMU04150, is positioned on the periphery of a clade containing both Hor and Hop proteins of *H. pylori *(Fig. 2). However HMU04150 lacks the characteristic (AEX [D, N]G) motif present in the *H. pylori *Hop proteins, and its carboxy terminal motif (Table 3) is more similar to Hor proteins. HMU11950 clusters with the three FecA orthologues of *H. pylori*. The majority of the remaining *H. mustelae *OMPs are currently unclassified (Table 3). They almost all share the properties of relatively small size, and lack of significant-identity database homologues. Some have atypical carboxy terminal sequences for OMPs, and signal peptidase cleavage sites that are not readily predicted. Two of them are expressed (see below), indicating significant production levels, and the biological function of these unclassified OMPs warrants further investigation.

**Table 3:** Classification of *H. mustelae *outer membrane proteins in relation to *H. pylori *OMPs

Gene	Protein length (AA)	Predicted MW (Da)	**N-terminal signal motif**^†^	C-term motif	FASTA Smith-Waterman identity*	Detected in proteome
**Hof-related OMPs**						
HMU05640	456	51,804	A?MDF	-YSF	32.1% in 479 aa overlap with H. pylori HofB (HP1083)	Yes
HMU05650	451	51,181	A?VET	-YGF	36.2% in 496 aa overlap with H. pylori HofF (HP0788)	Yes
HMU10680	447	50,681	A?ASS	-YFF	29.3% in 474 aa overlap with H. pylori HofE (HP0782)	No
**Hop-related OMP**						
HMU04150	189	20,863	R?AYD?	-YSF	34.7% in 170 aa overlap with HP0608	No
**FecA-related OMP**						
HMU11950	220	25,230	?	-ENP	No significant homologues	No
**Unclassified OMPS**						
HMU00140	232	26,345	G↓AGT	-FLF	No significant homologues	No
HMU00150	252	28,374	A↓KED	-IIF	No significant homologues	No
HMU00180	242	27,650	unclear	-YVF	No significant homologues	No
HMU00690	208	22,360	A↓EAI	-YTF	No significant homologues	No
HMU00810	135	15,492	A↓LVD	-IRY	No significant homologues	No
HMU01060	316	34,414	E↓ATK	-WYF	39.7% in 194 aa overlap with HomD (HP1453)	No
HMU02490	469	51,412	A↓GKL	-YNF	No significant homologues	No
HMU03240	394	32,308	A↓IEN	-FYF	34.5 in 281 aa overlap with HP0726	No
HMU03280	282	25,467	unclear	-KFF	No significant homologues	No
HMU03510	162	17,686	V↓SPE	-VEF	No significant homologues	No
HMU04930	899	98,821	A↓YNP?	-WVF	27.2% in 913 aa overlap with HomC (HP0373)	No
HMU07910	873	98,252	A↓NEI	-YHF	32.0% in 443 aa overlap with HofD (HP0487)	No
HMU09930	565	62,131	V↓AGA	-YHW	55% in 586 aa overlap with OmpP1 (HP0839)	No
HMU13480	258	28,279	A↓TKG?	-LIY	32.0% in 259 aa overlap with HH_1629	No
HMU13600	190	21,548	A↓MTL	-EAK	No significant homologues	Yes
HMU13670	173	19,128	A↓KTC	-NIK	No significant homologues	Yes
HMU13270	236	27,193	↓	-NHL	No significant homologues	No
HMU00260	196	21,989	A↓HAR?	-FVF	No significant homologues	No
HMU07990	343	39,155	A↓ENL	-FAF	42.9% in 233 aa overlap with HP0694	No
HMU10040	167	18,839	↓	-EGK	50.9% in 163 aa overlap with HP1546	No
HMU01230	232	24,626	A↓NTN	-YKF	44.5% in 236 aa overlap with HH_0525	No

† Predicted by SignalP 2.0 HMM with confidence, with low confidence (?), or not predicted (unclear).

*Only homologies with E-value <10^-10 ^presented.

The *H. mustelae *genome encodes many other proteins likely to contribute to virulence, based upon information available for homologues (Table 2). Close to the origin of replication, in a region distinguished by anomalously low GC content, are two genes related to haemolysis or haemagglutination. HMU00160 encodes a predicted protein with significant homology to haemolysin activators of diverse gram-negatives including *Photorhabdus luminescens*, *Burkholderia pseudomallei*, and a pathogenicity-island encoded determinant of *E. coli *[63]. HMU00170 Hag/Hly encodes a predicted 227 kDa protein with predicted signal peptide, and containing Pfam motifs for haemagglutination (Haemagg_act; PF05860), filamentous haemagglutinin (Fil_haemagg; PF05594), and an ATP/GTP-binding site motif A (P-loop). Homologues of this protein constitute a large family whose members are widely distributed among gram-negative pathogens, are annotated as either haemagglutinins or haemolysins, but which appear to lack functional characterization. The residue identity with HMU00170 is confined to the first 350 residues of that protein, and is particularly high over the filamentous haemagglutinin region (ca. 35-50% identity). Homologues of this pair of genes are lacking in helicobacters and campylobacters, suggesting this is an *H. mustelae*-specific acquisition among the ε-proteobacteria.

*H. mustelae *has two orthologs (HMU02820 and HMU09010) of HP0508, which has been characterized as a plasminogen binding protein in *H. pylori *[64]. The biological significance of this phenotype in either gastric pathogen is unclear. HMU0700 was annotated as CiaB by virtue of containing a low molecular weight phosphotyrosine protein phosphatase Pfam domain, and significant residue identity to the *C. jejuni *CiaB (Campylobacter Invasion Antigen B; Cj0914c) protein. This protein is required for maximal invasion of epithelial cells by *C. jejuni*, and is notably exported by the flagellar export apparatus [65]. Another recently discovered Cia protein, Cj1242 [66], is not present in the *H. mustelae *genome. Homologues of CiaB have been annotated in *W. succinogenes *and *H. hepaticus*, but not in *H. pylori*, which is curious because CiaB is also present in ε-proteobacteria isolated from sea vents [67], which are located on the deepest branch of the ε-proteobacterial tree. Like *H. pylori*, *H. mustelae *lacks the N-glycosylation system that contributes to pathogenesis in Campylobacters [68,69], and the genes for which are also present in *H. hepaticus*, *W. succinogenes *and sea vent ε-proteobacteria [67]. HMU10120 is a homologue of proteins in other Campylobacterales which was first described in *C. jejuni *as PEB4a, and which is a major antigen and cell adhesin [70,71]. The HMU10120 gene product was detected in the *H. mustelae *proteome (see below). Its role as an adhesin warrants further scrutiny, since it surprisingly contains a rotamase Pfam domain. Another candidate virulence/survival determinant in *H. mustelae *is HMU12690, which is a homologue of the *H. pylori *neutrophil activating protein HP0243 [72]. This has recently been described as one of three *H. pylori *proteins diagnostically predictive for development of gastric cancer [73]. NapA also has a role in protecting *H. pylori *from oxidative stress [74]. These features, coupled with the high-level expression of the HMU12690 protein in *H. mustelae *(see below), suggest that it may be relevant for survival or pathogenesis in the ferret stomach. The *H. mustelae *gene HMU06150 is homologous to Cj1327 and Cj1328, two genes involved in sialic acid biosynthesis, HMU06140 is annotated as an acylneuraminate cytidylyltransferase. Thus, *H. mustelae *may decorate its surface with sialic acid.

**Figure 3:**
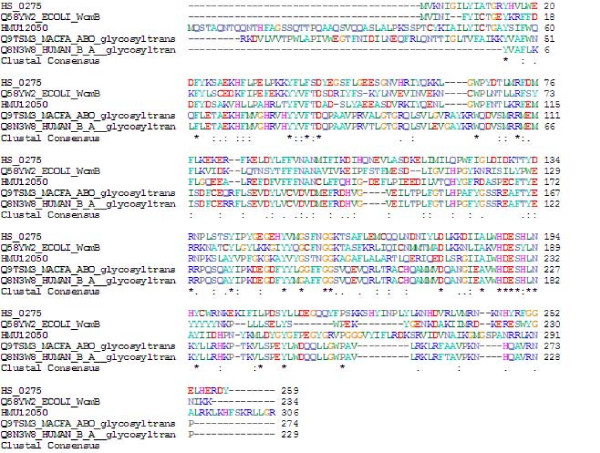
**ClustalW alignment of the HMU12050-encoded protein with homologues in *Haemophilus somnus*; *E. coli *(WcmB), *Macaca fascicularis *ABO glycoysltransferase; *Homo sapiens *AB glycosyltransferase**.

*H. pylori *incorporates human blood group antigens into its LPS (reviewed in references [75-77]) in a strain dependent manner. Ferrets express a structure equivalent to human blood group A on gastric tissue, and *H. mustelae *strains express blood group A antigen in their LPS [47,78]. The *H. mustelae *genome includes divergent orthologues of ten out of fourteen genes [77] implicated in *H. pylori *LPS biosythesis/blood group antigen production (Additional File 2). Some of these candidate orthologues are so divergent that they cannot be confidently separated from potential flagellin glycosylation genes (see below). The *H. mustelae *repertoire includes a single predicted fucosyl transferase, encoded by HMU12060. *H. pylori *fucosyltransferases display low identity to mammalian enzymes. Interestingly, HMU12050 encodes a predicted blood group AB glycosyltransferase, which shows 31-33% BLAST identity against mammalian AB glycosyltransferases, and putative glycosyltransferases from *E. coli *O86 and *Haemophilus somnus *(Fig. 3). To our knowledge, this is the first identification of a bacterial gene for synthesizing mammalian blood group A/B antigen which is known to be actually produced on the bacterial surface. It is expected by analogy with *H. pylori *(reviewed in ref. [75]) that this gene product will contribute to the ability of *H. mustelae *to adapt to the gastric environment (immune avoidance), modulate inflammation and immune cell recognition, and exacerbate pathology by triggering autoimmunity. The ferret model provides an excellent platform to test these hypotheses.

There are 4 secretion systems predicted in the *H. mustelae *genome (see below), including the flagellum protein export system. Motility conferred by flagella is an essential property for successful colonization of the ferret by *H. mustelae *[79], and the hook and flagellin proteins of *H. mustelae *have already been characterized [45,46]. The annotation of the *H. mustelae *genome revealed a typical set [80] of Campylobacterales flagellar genes (Additional File 3), for structural components, glycosylation, regulation, and chemotaxis. The number of chemotaxis genes is reduced compared to *H. pylori*, with orthologs of *cheV1*, *tlpC*, and *tlpA *apparently being absent. This may be functionally offset by the presence of HMU05990, a putative MCP-type signal transduction protein, which includes a PAS domain sensor sequence (Pfam 08447). This protein is absent in *H. pylori *and *H. hepaticus*, and its closest homolog is in *C. jejuni*. *H. pylori *contains several genes including HP0840 and HP0366, whose products result in glycosylation of flagellin with pseudaminic acid [81], which is required for flagellin assembly into flagellar filaments [82]. The *H. mustelae *genome includes a clear orthologue of HP0840 (designated *flaA1*; Additional file 2, Table S2). Two potential homologues of HP0366, HMU06610 and HMU02370, were identified in the *H. mustelae *genome. Another group of *H. mustelae *genes, HMU11700-HMU11730, shows some relatedness to Cj1311-Cj1317, involved in flagellin sialylation, but their function, and indeed the glycosylation state of *H. mustelae *flagellins, is still unknown. A noteworthy feature is the fact that two essential motility genes, *fliK *(HMU07800; hook length control protein) and *motA *(HMU03580; motor protein) are pseudogenes in the sequenced strain, which we subsequently confirmed to be non-motile (data not shown). The original type strain used for the species description emendation [17] was motile, as are *H. mustelae *isolates from wild ferrets [18]. In the case of FliK, HMU07800 is flanked upstream by an ORF encoding 277 amino acids, which is preceded by a perfect GG-N_10_-GC σ^54 ^promoter motif expected for *fliK *[83,84]. Thus, a frame-shift between HMU07800 and HMU07790 has inactivated *fliK*. The gene for MotA also appears to have suffered a frameshift. We assume that these mutations occurred during recent laboratory passage, in a manner similar to frame-shift inactivation of *fliP *in *H. pylori *strain 26695 [85], revertants of which can be easily obtained on motility agar at high plating density.

The 3 other complete or partial protein secretion systems predicted from the *H. mustelae *genome are presented in Additional File 4. The Sec system genes are not linked, except for *secD *and *secF*, which are clustered with *yajC*. Like other ε-proteobacteria, *H. mustelae *lacks SecB; it has a single *secA *gene. The *secE *gene was found by homology search, internal to the *tlp *gene and in the opposite strand. There is a single *tatB-tatC *gene cluster. The *tatA *gene is present (HMU02290) but the *tatE *gene is apparently absent. It is thus not clear if the *H. mustelae *Tat system is functional. Relevant for the abundance of autotransporters, we annotated a gene predicted to encode an Omp85(YaeT) homolog, which has a critical role in outer membrane protein insertion/biogenesis [86]. Analysis of the Gsp genes suggested the presence of a fragmented or remnant pilin biosynthesis system. The genes encoding GspDEF (also called CtsDEF) are clustered. However, there are other ORFs around them that have no significant homology to Gsp or type IV pilin-related proteins, except for a putative pseudopilin but this is unusually distantly separated from the others. Putative PilT-encoding and prepilin peptidase genes were also found separately on the chromosome, and not near anything that looks like encoding type IV pili or GSP machinery. Thus there may be a pilin assembly unit in the *H. mustelae *genome, which could contribute to pathogenicity, but functional investigation is required. Types IV and VI secretion system components were not found.

The presence of homopolymeric tracts in and between genes has been identified as a potential antigenic variation mechanism in *C. jejuni *[31], *H. pylori *[28] and *H. hepaticus *[29], and has been postulated to compensate for the relative paucity of transcriptional regulators. Disregarding polyA or polyT repeats because of the high genomic AT content, we identified 12 genes potentially affected by variation in copy number of intragenic homopolymers, and 8 potentially affected by intergenic variation (Additional file 5). Only two of the former category showed actual length variation in the shot-gun read data, compared to three of the latter. As expected from other Campylobacterales, the dominant gene function affected was surface architecture, at either protein or carbohydrate level. However, the overall number of genes potentially affected by this putative method of antigenic variation was significantly lower than *H. pylori, C. jejuni *or *H. hepaticus*. This may be due to the dominant coverage by the Hsr protein, which is a major antigen, and which changes epitopes by recombination [44].

### The Expressed Proteome of *H. mustelae*

**Figure 4:**
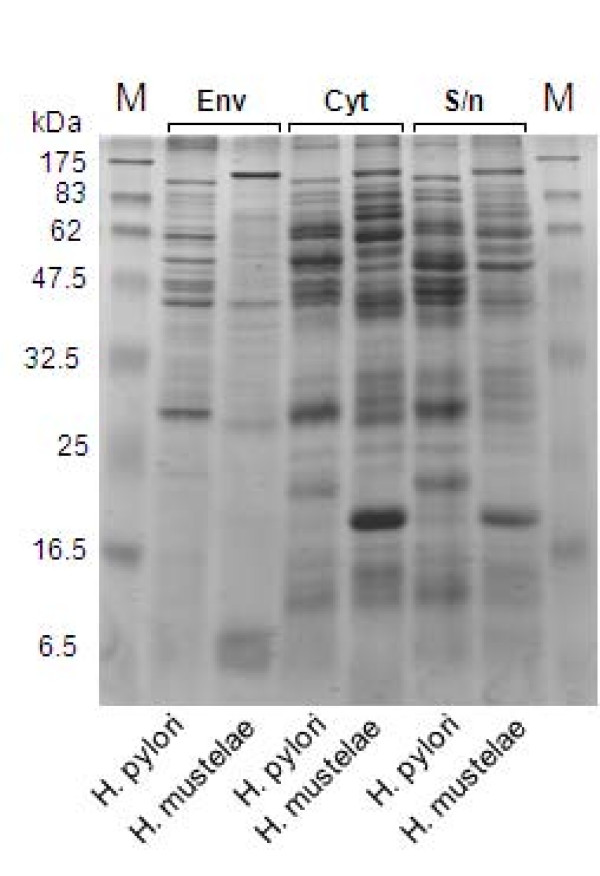
**SDS-PAGE analysis of *H. pylori *and *H. mustelae *sub-cellular fractions**. M: Prestained protein markers of indicated size. Env, cell envelope fraction. Cyt, cytosolic fraction.

We prepared sub-cellular fractions from *H. pylori *and *H. mustelae*, and first compared them by SDS-PAGE (Fig. 4). We cultured both species for two days on plates, compared to five-days used for the initial *H. pylori *proteome analysis [87], to minimize development of coccoid forms [88]. The initial supernatant from harvesting the cells was designated as an extracellular fraction, since it was expected to contain exported proteins. In accordance with the well-documented property of autolysis for *H. pylori *[89], the extracellular fraction of both species shared many bands with the cytosolic fraction of the respective species (Fig. 4). However, it was also clear that most of the proteins were apparently not shared between the two species. The greatest number of co-migrating bands between species was observed in the cytosol fraction, while the envelope fractions of the two species contained distinctive protein profiles. The *H. mustelae *envelope fraction contained around eight major proteins, less than half the number in the *H. pylori *envelope fraction, and few if any appeared to be produced by both species, consistent with the predictions from their respective genome sequences.

The dominant proteins in the envelope and cytoplasmic compartments of *H. mustelae *were identified by LC-MS. The most abundant 50 proteins in each fraction are presented in Table 4 and Table 5; the complete datasets are available in Additional file 6 and Additional file 7. The membrane proteome includes several cytoplasmic proteins that are also known to be highly expressed in *H. pylori*, including alkyl hydroperoxide reductase AhpC, flavodoxin and thioredoxin [87], and bacterioferritin [90]. Resistance to oxidative stress, and electron transfer functions, are clearly important processes that are performed using similar proteins in the two species. These proteins are all known to form either higher molecular weight aggregates, or membrane associations, which may explain their presence in the insoluble cell fraction. The AhpC protein, originally and mistakenly thought to be *H. pylori-*specific [91], was reported to be produced by several other *Helicobacter *species but not *H. mustelae *[92], although the gene was detectable in *H. mustelae *by PCR. The abundant soluble urease subunits A and B were also present in the insoluble fraction, as well as the cytosolic fraction, either through aggregation or membrane association in the former. The UreA2 and UreB2 structural sub-units were not detected, even though their mass fingerprints are clearly distinguishable from UreA and UreB (not shown). This non-production under our growth conditions is consistent with the observation that the expression of the Ure2 operon in *H. mustelae *only occurs under nickel limitation [41]. Despite the apparent lack of similarity between the *H. pylori *and *H. mustelae *proteomes in one-dimensional electrophoresis, when the 20 most abundant proteins detected in *H. pylori *by two-dimensional electrophoresis [87] were cross-compared to the *H. mustelae *cytosolic proteome, all 20 were present in the latter sample (Table 6). The relative abundances cannot be reliably compared due to differences in the methodologies, and growth phases of cells. The shorter growth period we used is reflected by the lower levels of stress proteins and higher levels of elongation factor EF-Tu in the detected *H. mustelae *proteome. Future comparative transcriptomic and proteomic investigations are needed to identify variations in core genome expression between the two species. In addition, we will compare the *H. mustelae *transcriptome and proteome after 5 days growth to that of *H. pylori*, to clarify comparative issues with the current datasets.

**Table 4:** The envelope proteome of *H. mustelae *determined by LC-MS

Rank^a^	Locus	Annotation	Mw Da	MOWSE Score^b^	Coverage^c^%	emPAIe^d^	Mol%
1	HMU14370	cft ferritin	19,056	455	53	2.72	5.74
2	HMU03320	ahpC alkyl hydroperoxide reductase	22,088	806	68	2.48	5.22
3	HMU12840	aroQ 3-dehydroquinate dehydratase	17,477	105	21	2.16	4.55
4	HMU14250	undefined product; COG4969, tfp pilus assembly protein, major pilin PilA [cell motility and secretion/Intracellular trafficking and secretion]	15,880	241	50	1.42	3
5	HMU00320	undefined product; no putative conserved domains detected, hypothetical protein Lreu23DRAFT_1924 [L. reuteri 100-23]: 35% ID	8,942	158	33	1.37	2.89
6	HMU14210	fldA flavodoxin 1	18,147	231	25	1.31	2.76
7	HMU03050	ureA fusion of urease beta and gamma subunits	25,199	683	60	1.15	2.43
8	HMU03060	ureB urease alpha subunit	61,146	1,218	37	1.048	2.21
9	HMU04000	groEL 60 kD chaperonin (cpn60)	57,443	1,714	51	1.035	2.18
10	HMU12690	possible bacterioferritin	17,198	380	32	1.015	2.14
11	HMU03500	putative amino-acid transporter periplasmic solute-binding protein	30,533	626	52	0.96	2.02
12	HMU01210	tpx probable thiol peroxidase	17,993	350	38	0.83	1.75
13	HMU00630	putative autotransporter protein	151,727	1,371	25	0.82	1.73
14	HMU13930	putative exported protein; COG1464, NlpA, ABC-type metal ion transport system, periplasmic component/surface antigen [inorganic ion transport and metabolism]	28,862	425	30	0.82	1.73
15	HMU01180	putative membrane-anchored cell surface protein	281,808	2,432	22	0.81	1.72
16	HMU03120	tuf elongation factor TU	43,570	850	48	0.77	1.64
17	HMU04030	petA putative putative ubiquinol-cytochrome C reductase iron-sulfur subunit	17,916	227	46	0.77	1.64
18	HMU06410	undefined product; No putative conserved domains detected, hypothetical protein Hac_1480 [Helicobacter acinonychis str. Sheeba]: 29% ID	20,229	254	18	0.77	1.64
19	HMU03990	groES 10 kD chaperonin (cpn10)	10,185	157	31	0.71	1.51
20	HMU09770	trxA thioredoxin	11,553	194	28	0.71	1.51
21	HMU08630	putative outer membrane autotransporter	155,228	968	14	0.64	1.37
22	HMU05030	putative hydantoinase A	78,444	785	30	0.54	1.15
23	HMU13940	putative exported protein; COG1464: ABC-type metal ion transport system, periplasmic component/surface antigen [inorganic ion transport and metabolism]	29,374	430	29	0.54	1.15
24	HMU05020	undefined product; COG4647/pfam08882, acetone_carb_G	14,279	213	29	0.49	1.04
25	HMU01190	putative hypothetical glycine-rich autotransporter protein	194,616	1,281	19	0.48	1.02
26	HMU00600	putative LPXTG surface protein	133,006	651	14	0.47	1
27	HMU09610	secG putative protein-export membrane protein	12,734	81	21	0.46	0.98
28	HMU12860	sodB superoxide dismutase (Fe)	24,576	231	23	0.46	0.98
29	HMU14090	putative thioredoxin	11,796	138	30	0.46	0.98
30	HMU11370	undefined product; no putative conserved domains detected, hypothetical protein Abu_2077 [Arcobacter butzleri RM4018]: 37%	7,677	92	26	0.46	0.98
31	HMU05010	putative hydantoin hydantoinase A	70,769	835	31	0.45	0.96
32	HMU10410	rpsH 30S ribosomal protein S8	14,807	247	32	0.43	0.91
33	HMU10080	putative putative membrane protein	10,902	103	17	0.42	0.89
34	HMU04360	atpC ATP synthase F1 sector epsilon subunit	13,211	108	16	0.42	0.89
35	HMU04350	atpD ATP synthase F1 sector beta subunit	51,310	581	23	0.42	0.89
36	HMU10380	rpsE 30S ribosomal protein S5	15,592	199	33	0.42	0.89
37	HMU09160	undefined product; no putative conserved hits detected, hypothetical protein HH1743 [Helicobacter hepaticus ATCC 51449]: 32% ID	43,936	335	14	0.40	0.85
38	HMU10290	rpoA DNA-directed RNA polymerase alpha chain	37,551	320	24	0.38	0.82
39	HMU05040	putative hydantoin utilization protein B	83,272	514	23	0.37	0.8
40	HMU05840	putative flagellin	53,982	501	27	0.37	0.79
41	HMU10120	peb4[1]f2 major antigenic peptide PEB3ll binding factor 2	31,816	371	32	0.35	0.76
42	HMU04050	petC putative ubiquinol-cytochrome C reductase cytochrome C subunit	33,425	299	25	0.35	0.74
43	HMU04380	exbD3 exbD olR family transport protein	14,662	132	23	0.35	0.74
44	HMU06080	hypothetical protein Cj0372; COG0754/pfarm03738: Glutathionylspermidine synthase [amino acid transport and metabolism]	44,843	197	12	0.35	0.74
45	HMU11040	fliL possible flagellar protein	18,741	175	18	0.33	0.7
46	HMU04330	atpA ATP synthase F1 sector alpha subunit	55,012	371	20	0.31	0.67
47	HMU07380	hupB DNA-binding protein HU homolog	10,122	142	26	0.29	0.61
48	HMU03350	rplS 50S ribosomal protein L19	13,546	226	28	0.29	0.61
49	HMU03180	rplL 50S ribosomal protein L7/L12	12,946	97	18	0.27	0.58
50	HMU01920	htrA serine protease (protease DO)	45,914	305	17	0.27	0.58

The 50 most abundant proteins are tabulated.

a. Relative abundance ranked by mol%

b. Score for the entire protein derived by MASCOT, and made up of the individual scores given to each peptide sequence

c. Proportion of each protein sequence identified

d. Exponentially modified protein abundance index. See Methods for details and reference.

**Table 5:** The cytosolic proteome of *H. mustelae *determined by LC-MS

Rank^a^	Locus	Annotation	Mw Da	MOWSE Score^b^	Coverage^c^%	emPAI^d^	Mol%
1	HMU14210	fldA flavodoxin 1	18,147	620	56	6.74	7.35
2	HMU05200	thiJ 4-methyl-5(beta-hydroxyethyl)-thiazole monophosphate synthesis protein	20,365	212	32	3.64	3.97
3	HMU01210	tpx probable thiol peroxidase	17,993	886	62	3.43	3.75
4	HMU12840	aroQ 3-dehydroquinate dehydratase	17,477	161	21	2.98	3.25
5	HMU12860	sodB superoxide dismutase (Fe)	24,576	534	45	2.41	2.63
6	HMU00320	undefined product	8,942	162	54	2.16	2.36
7	HMU05470	acpP acyl carrier protein	8,469	139	48	2.16	2.36
8	HMU03120	tuf elongation factor TU	43,570	1695	62	2.09	2.28
9	HMU12690	possible bacterioferritin	17,198	562	46	1.99	2.17
10	HMU09770	trxA thioredoxin	11,553	361	36	1.89	2.06
11	HMU04180	hypothetical protein Cj1613c	30,321	755	29	1.71	1.87
12	HMU03320	ahpC alkyl hydroperoxide reductase	22,088	572	44	1.68	1.83
13	HMU14370	cft ferritin	19,056	276	45	1.42	1.55
14	HMU00950	rpsA 30S ribosomal protein S1	60,196	208	7	1.31	1.43
15	HMU10800	lpsJ succinyl-CoA:3-ketoacid-coenzyme A transferase subunit B	23,051	501	38	1.15	1.26
16	HMU04000	groEL 60 kD chaperonin (cpn60)	57,443	1474	48	1.02	1.11
17	HMU03150	rplK 50S ribosomal protein L11	12,586	473	48	0.90	0.99
18	HMU01260	ald alanine dehydrogenase	39,844	662	32	0.82	0.9
19	HMU07380	hupB DNA-binding protein HU homolog	10,122	352	56	0.81	0.89
20	HMU10120	peb4[1]f2 major antigenic peptide PEB3ll binding factor 2	31,816	750	39	0.77	0.85
21	HMU07050	rplI 50S ribosomal protein L9	16,251	460	49	0.74	0.81
22	HMU04730	oorB OORB subunit of 2-oxoglutarate:acceptor oxidoreductase	30,613	423	28	0.74	0.81
23	HMU14090	putative thioredoxin	11,796	267	39	0.730	0.8
24	HMU04740	oorA OORA subunit of 2-oxoglutarate:acceptor oxidoreductase	40,773	554	36	0.70	0.77
25	HMU02170	cheY chemotaxis regulatory protein	13,894	208	25	0.70	0.76
26	HMU11060	putative putative aminotransferase (nifS protein homolog)	43,365	424	26	0.66	0.73
27	HMU00290	fbp putative putative fructose-1,6-bisphosphatase	30,550	323	20	0.66	0.73
28	HMU03180	rplL 50S ribosomal protein L7/L12	12,946	259	44	0.66	0.73
29	HMU00100	putative putative acyl-CoA thioester hydrolase	16,960	168	20	0.63	0.7
30	HMU13330	undefined product	8,555	166	49	0.63	0.7
31	HMU03850	flaG possible flagellar protein	14,814	452	34	0.62	0.69
32	HMU03060	ureB urease alpha subunit	61,146	743	26	0.62	0.68
33	HMU09760	trxB thioredoxin reductase	33,972	587	35	0.62	0.68
34	HMU07390	ndk nucleoside diphosphate kinase	15,293	291	25	0.61	0.67
35	HMU05040	putative hydantoin utilization protein B	83,272	948	28	0.60	0.66
36	HMU10790	scoA succinyl-CoA:3-ketoacid-coenzyme A transferase subunit A	25,076	305	32	0.58	0.64
37	HMU03990	groES 10 kD chaperonin (cpn10)	10,185	129	34	0.58	0.64
38	HMU13450	hypothetical protein Cj0706	27,047	694	49	0.55	0.61
39	HMU10960	katA catalase	52,490	781	39	0.53	0.59
40	HMU03670	aspA aspartate ammonia-lyase	51,394	620	31	0.51	0.57
41	HMU10290	rpoA DNA-directed RNA polymerase alpha chain	37,551	308	25	0.50	0.55
42	HMU11440	putative putative nucleotide phosphoribosyltransferase	17,378	164	25	0.50	0.55
43	HMU10630	putative putative periplasmic protein	21,100	282	24	0.48	0.53
44	HMU05030	putative putative hydantoinase A	78,444	681	24	0.47	0.52
45	HMU11200	glyA serine hydroxymethyltransferase	45,885	461	24	0.48	0.52
46	HMU08860	hypB hydrogenase isoenzymes formation protein	26,838	328	28	0.46	0.51
47	HMU01430	putative putative periplasmic cytochrome C	10,944	178	36	0.46	0.51
48	HMU06880	yabJ putative putative regulatory protein	13,506	163	26	0.46	0.51
49	HMU09820	surE SurE protein homolog	28,918	352	25	0.44	0.49
50	HMU12070	putative putative exported protein	16,551	270	33	0.44	0.49

The 50 most abundant proteins are tabulated.

a. Relative abundance ranked by mol%

b. Score for the entire protein derived by MASCOT, and made up of the individual scores given to each peptide sequence

c. Proportion of each protein sequence identified

d. Exponentially modified protein abundance index. See Methods for details and reference.

**Table 6:** Comparison of highly expressed proteins in the two dimensional electrophoresis pattern of *H. pylori *with abundant cytosolic proteins of *H. mustelae *detected by LC-MS

*H. pylori *ORF	Protein	Annotation^a^	*H. mustelae *ortholog	Rank in *H. mustelae *proteome^b^
HP0010	GroEL	Chaperone/heat-shock protein	HMU0400	16
HP0072	UreB	Urease β-subunit	HMU0305	66
HP1563	AhpC	Alkyl hydroperoxide reductase	HMU0332	12
HP0547	Cag26	Cag pathogenicity island protein	-	
HP0073	UreA	Urease α-subunit	HMU0306	32
HP1294	Rps4	Ribosomal proteinS4	HMU1030	120
HP1496	Ctc	S4 general stress protein	HMU0808	193
HP1199	Rpl7	Ribosomal protein L7	HMU0318	28
HP0390	TagD	Adhesin-thiol peroxidase	HMU0390	3
HP0011	GroES	Co-chaperone	HMU0399	37
HP0243	NapA	Neutrophil activating protein	HMU1269	9
HP1286	-	Conserved hypo. secreted protein	HMU0209	78
HP0570	PepA	Aminopeptidase A	HMU652	167
HP1205	TufB	Elongation factor EF-Tu	HMU0312	8

a. *H. pylori *annotation; b. Cytosolic fraction

The abundant members of the cell envelope proteome include proteins involved in metabolism (e.g. ATP synthase), transport (e.g. ABC transporter subunits), secretion (SecG, lower amounts of SecA), and several flagellar proteins (Table 4). Notable among the most abundant proteins is HMU14250, a hypothetical protein with homology to pseudopilin or pilin subunits (see above). Less than 1% of the expressed cytosolic proteome was annotated as "hypothetical". In contrast, six of the top fifty proteins in the membrane proteome were annotated as "hypothetical", as was 10% of the total detected membrane proteome, validating the gene annotation process, and highlighting the possible contribution of proteins of unknown function to the biology of *H. mustelae*. Of the 26 predicted outer membrane protein in *H. mustelae*, only 4 of these, HMU0564, HMU0565, HMU1360 and HMU1367, were detected in the membrane proteome (Table 4). The fact that two of these are encoded by contiguous genes and likely co-transcribed is suggestive that their successful detection is due to similarly high expression levels. It is likely that some or many of the other predicted outer membrane proteins are actually expressed, but are below the detection limit, estimated to be in the micromolar range. Surface proteins detected in the expressed proteome also included HMU04120, a putative OM component of an efflux system. Of the 10 autotransporter proteins annotated, 7 of these were detected, at relatively high levels. Interestingly, at 1.37 Mol%, the dominant surface ring-forming protein Hsr was not the most highly expressed protein. HMU0118 was detected at 1.72% Mol% and HMU0063 at 1.73%. HMU0118 is 29% identical to the Hsr protein and HMU0063 is 38.8% identical to Hsr, but in both cases, the identity at the amino terminal exposed part of the molecule is low. Although the Hsr gene was identified and cloned by immunoreactivity with antiserum raised against purified Hsr protein, and this antiserum labeled the surface rings by immunoelectron microscopy [42], the possibility remains that the surface rings are composed of more than one autotransporter protein. This would contribute to even greater antigenic variability of the *H. mustelae *surface caused by recombination of sequences for new epitopes into the expressed Hsr protein [44].

### Sequence motifs associated with high-level protein production

Relative protein production levels determined by high-throughput LC-MS will be modulated by factors including transcription rates, translation efficiency, susceptibility to proteolysis, and limitations of solubility. Notwithstanding these complexities, we searched the intergenic regions of the *H. mustelae *genome for motifs associated with the genes for the 426 proteins detected in the combined proteome fractions. Only non-coding intergenic regions, from positions -40 to -200 from the start codon were searched, as most DNA-binding motifs are found within these regions [93]. This analysis identified several sequence elements strongly associated with elevated protein detection proportions. The top five variants of each of five motifs detected upstream of genes in the envelope and cytosol fraction proteomes are presented in Additional file 8 and Additional file 9. Motifs associated with the most highly expressed proteins in the membrane fraction (Motifs 1 and 2) tended to be localized 60-150 nt from the start of the ORF and might represent a binding site for a positive regulator. Motif 3 overlapped the presumptive ribosome binding site except in one case. None of the motifs appeared to correspond to a composite promoter configuration, and in the case of genes with known promoters (e.g. *flaB*; HMU07150), the expected σ^54^-dependent promoter was not found. Interestingly, the motif listed in Additional file 8, Table S8 for HMU01180 was found, in identical sequence and position, on the opposite strand upstream of HMU01190. These two genes are essentially identical in the regions encoding the signal sequences and autotransporter domains, and could arguably have evolved by a gene duplication event, that would have conserved this motif without selection. Alternatively it could represent a conserved control element for these highly expressed proteins. The motifs located in the upstream flanks of the genes for highly expressed soluble proteins are also located further from the ORF, in most cases, than would be expected for a promoter. Motif 4, as for Motif 3 for the membrane proteins, is the Shine-Dalgarno sequence. Genome-wide functional analysis of helicobacter expression signals, by transcript mapping, deletion analyses and gene fusions, is warranted.

### Comparative Genomics and phylogeny of the ε-Proteobacteria

**Figure 5:**
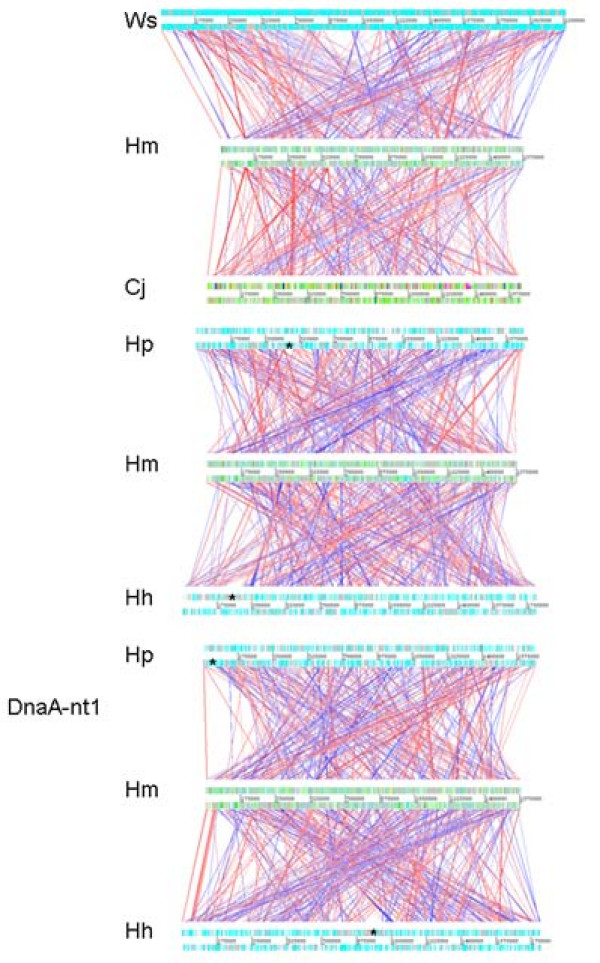
**Artemis Comparison Tool (ACT) alignment of selected genomes with that of *H. mustelae***. Alignments were performed as described in Methods and visualized with a 500 residue cut-off. In the bottom alignment, the published genome sequences of *H. pylori *and *H. hepaticus *were re-ordered so that co-ordinate1 was the start of the DnaA gene. The *cag *pathogenicity and genome island of *H. pylori *and *H. hepaticus*, respectively, are asterisked.

Alignment of the *H. mustelae *genome sequence with those of *W. succinogenes*, *C. jejuni*, *H. pylori *and *H. hepaticus *revealed lack of extensive i.e. long-range synteny with any of these genomes (Fig. 5), a feature noted for other genomic comparisons within the Campylobacterales [35]. Although the ACT software allows visualization of mutually reversed homologous sequences, it was noteworthy that comparing *H. mustelae *to *W. succinogenes *or *C. jejuni *seemed to more clearly highlight a vestigial genome backbone than comparing it to *H. pylori *or *H. hepaticus *(Fig. 5). Re-orienting the *H. pylori *and *H. hepaticus *genomes with the *dnaA *genes at co-ordinate 1 partly clarified the rungs of a conserved ladder of homology between the genomes, but this was still largely obscured by the numbers of relative transpositions and reversions of multiple loci between the genomes. The rungs in the ladder are formed by genes including *dnaA*, gyrase, a putative metallo-beta-lactamase (shared with *H. hepaticus*), and *gatB*, the 2-oxoglutarate:acceptor oxidoreductase operon. This analysis also highlighted the degree to which lack of synteny in the compared genomes is due to transposition across the origin-terminus axis, resulting in an X-shaped alignment that is symmetric about the origin of replication as previously noted in other bacteria by Eisen [94]. This symmetry indicates homologous loci at the same distance from the origin but on the opposite side of the origin, which is explained by the fork replication theory [95]. The genome alignments highlight the absence of the *H. pylori cag *pathogenicity island and the *H. hepaticus *genomic island (from HH_232 to HH_303) in the *H. mustelae *genome (Fig. 5). Relatively few longer stretches of the *H. mustelae *genome lack any significant homologues in *H. pylori *or *H. hepaticu*s; those that exist include HMU00600-HMU00690 that includes AT cluster 1; HMU01180-HMU01200 including AT cluster 2; and HMU10860-HMU10880 that encodes a predicted tricarboxylate transport system not found in the other *Helicobacter *species.

**Figure 6:**
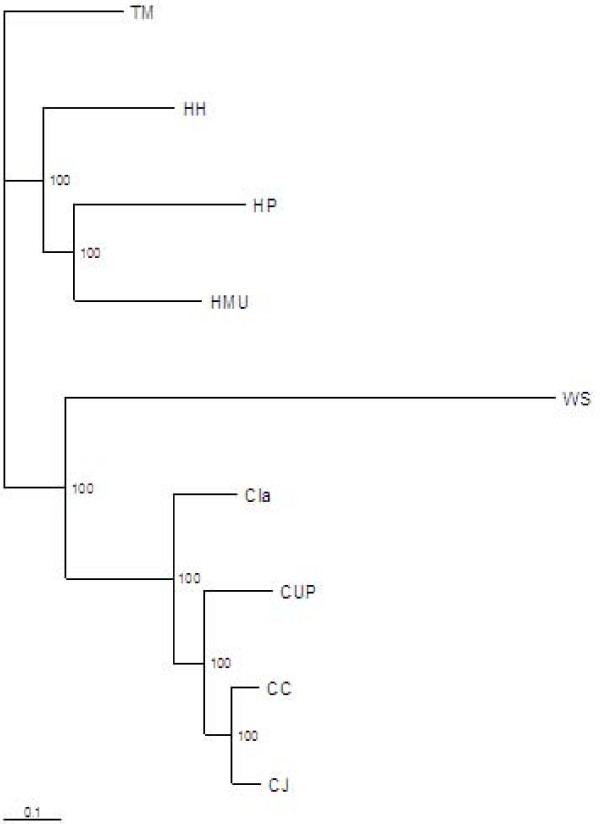
**Concatenated sequence-based phylogeny of selected *Helicobacter *and *Campylobacter *species**. Internal numbers correspond to bootstrap values. HMU: *H. mustelae*; HH, *H. hepaticus *; HP, *H. pylori; *WS, *W. succinogenes*; CUP, *C. uppsaliensis*; CLA, *C. lari*; CC, *C. coli*; CJ, *C. jejuni*.

To explore the phylogenomics of the *Campylobacterales *for which genome sequence data were available, we first performed pair-wise alignments of their proteomes and then constructed a matrix based on their relatedness. Using methods derived during our studies of another bacterial group within which genetic distances are very long, the *Lactobacillales *[96], we defined orthologues at protein level, requiring 30% identity over 80% of the sequence lengths. The pair-wise alignment data is presented in Additional file 10. These data indicated that *H. mustelae *was closest phylogenetically to *H. hepaticus*, followed by *H. pylori *and the the Campylobacters. A tree constructed based on 212 orthologous proteins shared between the respective taxa (Fig. 6) showed two major branches, one including the four *Camplyobacter *genomes. In the second branch, *H. mustelae *clustered most closely with *H. pylori *and with the enterohepatic species *H. hepaticus*. *W. succinogenes *was peripheral to the Campylobacter clade. This topology is more concordant with the 16S rRNA phylogeny of Dewhirst [26] and Gueneau [25] than with the 23S rRNA gene phylogeny constructed by Dewhirst *et al*, which the authors suggested to be more robust [26]. The positioning of *W. succinogenes *in particular by the 23S rRNA gene phylogeny is significantly different from that based on numbers of orthologs in the current study including *H. mustelae*. Our exclusion of *W. succinogenes *from the helicobacters also conflicts with a phylogeny constructed in the same study by Dewhirst and colleagues [26], based on 870 shared proteins.

**Figure 7:**
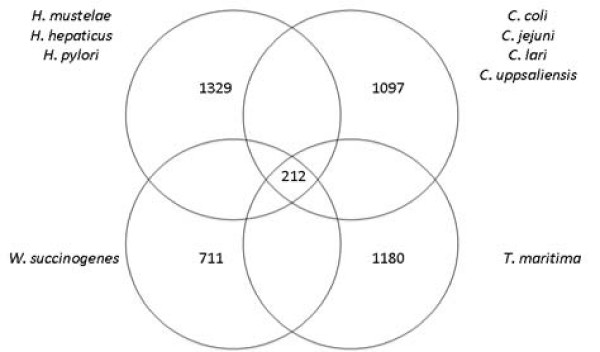
**Venn diagram showing numbers of orthologous proteins for genera within selected *Campylobacterales *whose genome sequences were analyzed**.

**Figure 8:**
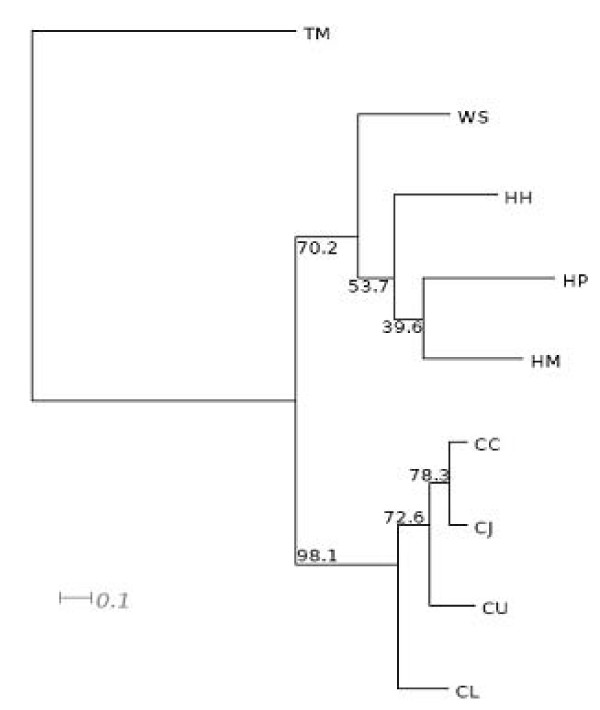
**Concatenated sequences-based phylogeny of selected *Helicobacter *and *Campylobacter *species**. Internal numbers correspond to bootstrap values. HMU: *H. mustelae*; HH, *H. hepaticus *; HP, *H. pylori*; WS, *W. succinogenes*; CUP, *C. uppsaliensis*; CLA, *C. lari*; CC, *C. coli*; CJ, *C, jejuni*.

We have previously used Supertree analysis to clarify relatedness in distant taxa [96]. The advantage of this approach is that it combines the maximum likelihood trees constructed from each of hundreds of core proteins, in this case the 212 proteins shared by all taxa (Fig. 7). This all-against-all comparison identified numbers of proteins specific to major groups (Fig. 7). The three helicobacters constituted a reasonably robust group, with over thirteen hundred core proteins, compared to 1097 in the four campylobacters. The consensus supertree constructed for the eight *Campylobacterales *plus outgroup is presented in Fig. 8. Based on this more restricted set of core proteins, *W. succinogenes *still positioned on the edge of the *Helicobacter *clade. *H. pylori *was most closely related to *H. mustelae*. However this *H. pylori*-*H. mustelae *branch was the least supported by the combined frequencies of the individual maximum likelihood trees, indicating the instability of this phylogenetic relationship. Considering the pairwise comparisons, whereby *H. mustelae *was most closely related to *H. hepaticus *(Additional file 9, Table S9), the choice of proteins clearly has a profound affect on the phylogeny inferred. The choice of *T. maritima *as outgroup may also have affected the outcome, but the number of shared orthologs was only 252 when this taxon was not included in the all-against-all comparison, suggesting this was not a major factor. As for the pair-wise ortholog analysis and the phylogeny based on concatened core proteins (Fig. 6), *W. succinogenes *did not cluster among the helicobacters, and the data do not support the notion of revising the nomenclature of these genera [26].

## Conclusions

*H. pylori *infects over half the global population [97]. Although the majority of infected individuals do not develop cancer [98], the prevalence rates mean that a significant number of subjects will suffer a preventable neoplastic transformation. Development of mammalian cancer in response to bacterial infection is a relatively recently recognized phenomenon [99], and this outcome has also been robustly attributed to *H. hepaticus *and *H. mustelae*. The *H. mustelae*-ferret system presents a model for studying the development of gastric cancer. Chronic inflammation is a risk factor for cancer, because of a shift in the balance of pro- and anti-apoptotic genes towards a more anti-apoptotic phenotype [100]. Human infections with *cag*-positive strains of *H. pylori *are much more frequently associated with neoplastic outcomes [101]. C*ag*-minus strains are less infrequently associated with non-atrophic gastritis and duodenal ulceration [102]. However it has long been recognized that infection with Cag-minus *H. pylori *poses a significantly greater risk for gastric cancer compared to non-infection [103]. Indeed in a recent primary study and accompanying meta-analysis, the increased gastric cancer risk due to infection by a Cag-minus strain compared to non-infection was four-fold [104], and the reviewed values in well controlled studies ranged from two-fold to five-fold. Given the time-scale over which neoplastic transformations occur, the development of cancer due to *Helicobacter *spp. will likely have a major component of chronic inflammation. Among the three species known to be carcinogenic, convergent infection-related disturbances of pro-apoptotic pathways is thus likely to be the key feature, rather than a single pro-carcinogenic microbial product or metabolite common to all three species. The strong linkage of *cag *to human gastric cancer thus appears to be a specific host-bacterium phenomenon. A search for "carcinogenicity determinants" should encompass complex persistence mechanisms and host-interaction molecules, and not focus solely on overt effectors such as CagA. Animal model systems may be useful for such searches. Haas and colleagues identified 47 *H. pylori *genes that were required for gastric colonization of the Mongolian Gerbil [105]. Orthologues of all but three of these genes are found in the *H. mustelae *genome (Additional file 11), including a collagenase shown to be a functional enzyme when cloned from *H. pylori *[105]. Some of the *H. mustelae *orthologues are quite divergent, suggesting a degree of host-adaptation or lack of strong selection, but nevertheless indicating that the primary gastric persistence mechanisms of *H. pylori *and *H. mustelae *are essentially conserved.

In addition to the lack of *cag *in *H. mustelae*, the lack of VacA and the major *H. pylori *adhesins indicates that colonization and persistence of these two gastric species is mechanistically different. This may simply reflect immunological and physiological differences between ferret and human stomach [106], but also the fact that the pathology of human gastritis is generally more severe [13]. The striking abundance of autotransporter proteins in *H. mustelae *strongly indicates a role for these molecules in host interaction. The Hsr AT protein decorates the *H. mustelae *surface in a paracrystalline array of ring structures that are antigenically variable, presumably to avoid a humoral immune response that would clear the infection. Attempts to develop vaccines against *H. pylori *have been largely unsuccessful, despite promising animal trials (reviewed in ref. [107]). The reasons for this failure are complex, but include the fact that key adhesins are low copy number OMPs, and that immune cells are actively targetted by VacA. Investigation of how the ferret immune response may be exploited to eradicate or prevent *H. mustelae *infection could be informative for preventing *H. pylori *infection in humans.

Paradoxically, *C. jejuni *persists as a commensal of birds, and *W. succinogenes *as a commensal of ruminants, despite both genomes being endowed with known (*C. jejuni*) or inferred (*W. succinogenes*) virulence determinants. As the sequencing of its genome has revealed, some of these are also shared with *H. mustelae*. Just as has been argued for *H. pylori *strains [2], and for pathogens in general [108], what defines a pathogen and a disease outcome involves the phenotype of the microbe, the genotype of the host, and the vigour and appropriateness of the host response. Comparative and functional genomics of the ε-proteobacteria will be a fertile area to explore these hypotheses.

## Methods

### Cell culture and growth conditions

*Helicobacter mustelae *strain 12198 (identical to CCUG 25175 and ATCC 43772, the type strain of *H. mustelae*) was cultured as described previously [87,109] on chocolate blood agar plates (CBA; Oxoid Basingstoke, Hampshire, UK) for 48 h at 37°C in an atmosphere containing 5% CO_2_.

### DNA extraction, genome sequencing, and annotation

High molecular weight genomic DNA of *H. mustelae *was extracted as previously described [42]. The genome of *H. mustelae *strain 12198 was sequenced to approximately 8-fold coverage, from pUC18 (insert size 2.8-3.3 kb and 3.0-3.3 kb) genomic shotgun libraries using big-dye terminator chemistry on ABI3730 automated sequencers. End sequences from large insert BAC libraries in pBACehr (insert size 10-25 kb) and pBACe3.6 (insert size 12-15 kb) were used as a scaffold. All repeat regions were bridged by read-pairs or end-sequenced polymerase chain reaction (PCR) products. The sequence was finished to standard criteria [110]. Sequence assembly, visualization, and finishing were performed using PHRAP (http://www.phrap.org; P. Green, unpublished data) and Gap4 [111]. The sequence and annotation of the *H. mustelae *12198 genome has been deposited in EMBL/GenBank/DDBJ under accession number FN555004.

The *H. mustelae *genome sequence was annotated using Artemis software [112]. Initial coding sequence (CDS) predictions were determined by Orpheus [113], Glimmer2 [114], and EasyGene software [115]. These predictions were collated and combined, and were further refined by reference to codon usage, positional base preference methods and comparisons to the non redundant protein databases using BLAST [116] and FASTA [117]. The entire DNA sequence was also compared in all six potential reading frames against UniProt, using BLASTX [116] to identify any possible coding sequences previously missed. Protein motifs were identified using Pfam [118] and Prosite [119], transmembrane domains were identified with TMHMM [120], and signal sequences were identified with SignalP version 2.0 [121]. rRNAs were identified using BLASTN [116] alignment to defined rRNAs from the EMBL nucleotide database; tRNAs were identified using tRNAscan-SE [122]; stable RNAs were identified using Rfam [123].

The *H. mustelae *genome was visualized in circular format using Genomeviz [124]. The input MAP file was produced from the corresponding Artemis file. COGS were assigned on the basis of a BLASTP comparison to an in-house COG database. GC % and GC skew maps were also generated in Genomeviz, with a window size of 1000 bp, and an overlap of 500 bp between windows.

Regions of the genome likely acquired by horizontal gene transfer were identified using the Alien Hunter algorithm [125] which works by finding local compositional biases based on a variable-order motif distributions method [125].

Motif searches, for detecting conserved motifs upstream of highly expressed genes, were performed using MEME [126], searching positions -40 to -200 of start codons. If the intergenic region was less that 40 nt, the downstream ORF in question was considered to be part of a operon and the intergenic region upstream of the first gene in the operon was selected for analysis. MEME was instructed to search the given strand (coding strand) for motifs between 6 and 50 bp in length and only on the coding strand. The MEME statistical parameter zoops (Zero or one Occurrence per Sequence) was set in the run command. Only motifs with an E value of <0.001 were considered relevant [126]. Weblogos summarizing the consensus motifs were derived using MEME. The E-value of a motif was defined as the number of motifs as good as or better than the motif in question which would appear in a random set of sequences the same size as the training set. The P-value is the probability that a random string will have the same score as the current one, and is thus an indication of the degree of similarity a string has to the consensus.

### Comparative genomics

Outer membrane protein phylogeny was investigated by first aligning a combined dataset of *H. pylori *26695 and *H. mustelae *predicted OMPs in MUSCLE [127], using the phylogeny.fr web server [128]. The aligned sequences were then used to construct a maximum likelihood phylogeny with Phyml [129]. Branch support values were calculated using the Approximate Likelihood Ratio test or aLRT) [130].

Sequence data for the *in silico *analyses were obtained from the NCBI reference sequences (RefSeq) for the circularized genomes of *Helicobacter hepaticus *ATCC 51449 (NC_004917), *H. pylori *26695 (NC_000915), *Campylobacter jejuni *subsp. *jejuni *NCTC 11168 (NC_002163), *Wolinella succinogenes *DSM 1740 (NC_005090) and *Thermotoga maritima *MSB8 (NC_000853) and for the incomplete genome sequences of *C. upsaliensis *RM3195 (AAFJ01000001), *C. lari *RM2100 (NC_012039) and *C. coli *RM2228 (AFL01000001). Proteome sets consisted of the translated gene sequences of respective complete or incomplete genomes.

Whole genomes were aligned using the Artemis Comparison Tool (ACT) [131]. Full genome sequence comparisons were performed using the BLAST program bl2seq. Comparisons were done at protein level with an e-value cut-off of 1E-08. Blast results were parsed with MSPcrunch and the resulting files visualized with ACT. In order to improve the visualization of synteny in the graphical alignments, the start regions of the *H. pylori *and *H. hepaticus *genome sequences were shifted to the beginning of their corresponding *dnaA *genes.

Proteome sets derived from respective complete or incomplete annotated bacterial genomes were compared pairwise, and all-against-all using BLASTP [116]. Our working definition of orthology was protein sequences that reciprocally shared more than 30% sequence identity using BLASTP over at least 80% of total sequence length, so that sets of pair-wise and all-against-all orthologs were obtained, correspondingly. The former set was used to build a pairwise comparison matrix, where the lower triangle indicates the total number of orthologs in genome-genome comparisons while the upper triangle shows the average sequence identity values expressed in percentage.

For the consensus tree, each of the 212 protein sequences were aligned using ClustalW, their best protein model of evolution was chosen, and a maximum likelihood tree was built for each protein set using Multiphyl [132]. Using these trees, a final consensus tree was built using the option ConsensusNetwork with a threshold of 0.33 using the Edge Weights mean option. To construct the concatenated phylogenomic tree, the 212amino acid sequences were concatenated for each organism. The resulting final sequences were aligned using Mafft v6.240 [133] with the option "auto". Columns in the resulting alignment with gaps in more than 50% of the sequences were deleted using Gblocks [134] in order to avoid poorly aligned positions and divergent regions. Using these sequences, a maximum likelihood tree was built using Phyml 3.0 [129] with default values and a bootstrap of 100 replicates. The resulting tree was visualized using TreeView [135], and in both consensus and concatenated trees *T. maritima *was used as an out-group.

### Protein extraction and quantification

All reagents were purchased from Sigma-Aldrich (Poole, UK) with the exception of mass spectrometry grade water and acetonitrile, which were purchased from Romil (Cambridge, UK) and trypsin, which was purchased from Promega (Southampton, UK).

Bacterial cells were harvested from CBA plates into phosphate buffered saline pH 7.4 (PBS; Sigma, Dorset, UK) and cell numbers were adjusted by the addition of PBS to obtain absorbance values of 0.5 (600 nm), determined by viable count to be equivalent to 4 × 10^8 ^CFU ml^-1^. A 20 ml volume of each cell suspension was centrifuged at 8,600 × g at 4°C for 30 min. Cell pellets were weighed and resuspended in 10 mM PBS (pH 7.8) at ratios of 1 g cells to 2 ml buffer. The cells were then broken using sonication as described previously by Graham *et al*. [136]. The soluble proteome fraction was isolated by centrifugation of the homogenate at 25,000 × g for 30 min at 3-5°C (Beckman J2-HS, Beckman Instruments, CA, USA) followed by ultracentrifugation at 150,000 × g for 2 hours at 3-5°C (Beckman L8-M, Beckman Instruments, CA, USA) to sediment insoluble fractions. Supernatant fractions were decanted and stored in 1 ml aliquots at -70°C until required. The insoluble fractions were weighed and resuspended in 2% SDS in PBS at ratios of 1 g cells to 2 ml buffer, treated in for 45 mins at 4°C, and then also stored in 100 μl aliquots at -70°C until required.

### One Dimensional Gel Electrophoresis

Protein concentrations were measured using the Bradford assay [137] and aliquots of supernatant and insoluble fractions were added to 10 μL Tris-Glycine SDS sample loading buffer (Invitrogen, Renfrewshire, UK), made up to 40 μl with dH2O, and boiled for 5 min. The samples (20 μL; 100 μg total protein) were loaded onto a 1 mm thick Nu-Page 4-12% Bis-Tris gel (Invitrogen, Renfrewshire, UK). SeeBlue™ Plus 2 (Invitrogen, Renfrewshire, UK) was used as a protein molecular mass marker. The gel was electrophoresed, using MES SDS running buffer, in an X-Cell II mini gel system (Invitrogen, Renfrewshire, UK) at 200 V, 120 mA, 25 W per gel for 35 min. Proteins were visualised using SimplyBlue™ Safestain (Invitrogen, Renfrewshire, UK). The entire lane was excised from the gel and cut into eight fractions based on molecular mass as previously described by Graham *et al*. [138,139]

### In-Gel Tryptic Digestion

Excised gel fractions were washed for 30 min in 200 mM NH_4_HCO_3_, pH 7.8 at 37°C. These fractions were then dehydrated by incubation for 30 min in 200 mM NH_4_HCO_3 _pH 7.8/MeCN (4:6 v/v) at 37°C, followed by rehydration for 30 min in 50 mM NH_4_HCO_3_, pH 7.8 at 37°C. Following incubation in 100% acetonitrile for 2 min, 0.1 μg trypsin in 50 mM NH_4_HCO_3_, pH 7.8 was added to each sample, which was then incubated overnight at 37°C. The supernatant was subsequently recovered into microcentrifuge tubes and a second peptide extraction from these gel pieces was carried out (0.1% TFA in 60% acetonitrile for 5 min). Peptide-containing liquid fractions were pooled, dried under vacuum and re-suspended in 20 μL 0.1% formic acid in 2% acetonitrile prior to storage at -70°C until required.

### Liquid Chromatography-Mass Spectrometric Analysis (LC-MS)

Mass spectrometry was performed using a 3200 Q-TRAP Hybrid ESI Quadropole linear ion trap mass spectrometer, ESI-Q-q-Qlinear ion trap-MS/MS (Applied Biosystems/MDS SCIEX, Toronto, Canada) with a nanospray interface, coupled with an online Ultimate 3000 nanoflow liquid chromatography system (Dionex/LC Packings, Amsterdam, The Netherlands). A μ-Precolumn™ Cartridge (300 μm × 5 mm, 5 μm particle size) was placed prior to the C_18 _capillary column (75 μm × 150 mm, 3 μm particle size) to enable desalting and filtering. Both columns contained the reversed phase material PepMAP™ 100 (C_18 _silica-based) with a 100Å pore size (Dionex/LC Packings). The elution buffers used in the gradient were Buffer A (0.1% formic acid in 2% acetonitrile) and Buffer B (0.1% formic acid in 80% acetonitrile). The nanoLC gradient used was 60 min in length: 0 - 55% B in 45 min, 10 min at 90% B followed by 5 min at 100% A. The flow rate of the gradient was 300 nLmin^-1^. The detector mass range was set at 400-2000 *m/z*. MS data acquisition was performed in positive ion mode. During MS acquisition peptides with^2+^and^3+ ^charge state were selected for fragmentation.

### Database Searching, Protein Identification and PROVALT Analysis

Protein identification was carried out using an internal MASCOT server (version 1.9; Matrix Science, London, UK) searching against the *H. mustelae *genome database. Peptide tolerance was set at ± 2.0 Da with MS/MS tolerance set at ± 0.8 Da and the search set to allow for 1 missed cleavage, and allowed for fixed modifications of carbamidomethylation and variable modifications of oxidation of methionine residues. In order to expedite the curation of the identified protein list from MASCOT, the result files were re-analysed against an extracted database comprising the *H. mustelae *file using the heuristic method known as the protein validation tool PROVALT [140]. This automated program takes large proteomic MS datasets and reorganises them by taking multiple MASCOT results and identifying those peptides that match. Redundant peptides are removed and related peptides are grouped together associated with their predicted matching protein, thus, the program dramatically reduces this portion of the curation process. For identification purposes the minimum peptide length was set at 6 amino acids, minimum peptide MOWSE score was set at 10 and the minimum high quality peptide MOWSE score was set at 22. PROVALT also uses peptide matches from a random database (in this case the extracted *H. mustelae *protein database was randomised) to calculate false-discovery rates (FDR) for protein identifications as previously described by Weatherley *et al*. [140]. Briefly, identifications from searching the normal and random databases are used to calculate the FDRs and set score thresholds and thus identify as many 'actual' proteins as possible while encountering a minimal number of false-positive protein identifications. Rather than calculate error rates at the peptide level, the FDR calculations employed by PROVALT provide a reasonable balance between the number of correct and incorrect protein assignments. In this study the FDR was set at 1%, meaning that 99% of the reported proteins identified should be correct. All detected proteins were then quantified by utilising the exponentially modified protein abundance index (emPAI) [139,141,142]. This method allows the quantification of individual identified proteins by utilising database and MASCOT output information (based on number of peptides identified), in order to give an emPAI value http://www.matrixscience.com/help/quant_empai_help.html. The emPAI values were then be used to estimate protein content within sample mixtures in molar fraction percentages as previously described [139,141,142].

## Authors' contributions

PWOT conceived of the study, participated in its design and coordination, analyzed the data and drafted the manuscript. WJS prepared sub-cellular fractions and analyzed them by 1DE and LC-MS. CC performed comparative genome analysis, phylogeny and phylogenomics. BMF performed genome sequence analysis. KRH and CJ annotated secretion system genes, and flagellar genes, respectively. RLJG and GMcM conceived the LC-MS analysis and analyzed proteome data. EB performed gene prediction and annotation. JP and SB designed and implemented the sequencing strategy, analyzed the data and drafted the manuscript. All authors read and approved the final manuscript.

## Supplementary Material

Additional file 1 Autotransporter loci in the *H. mustelae *genome. Click here for file

Additional file 2 Blood group antigen-associated genes in the genome sequence of *H. mustelae*, and compared with *H. pylori* Click here for file

Additional file 3 Motility- and flagellum-associated genes in the genome sequence of *H. mustelae*, and compared with *H. pylori* Click here for file

Additional file 4 Protein secretion-associated genes in the genome sequence of *H. mustelae*, and compared with *H. pylori* Click here for file

Additional file 5 Presence of homopolyeric tracts within/between *H. mustelae *genes, and homopolymer length variation in sequence data Click here for file

Additional file 6 The envelope proteome of *H. mustelae *determined by LC-MS. Click here for file

Additional file 7 The cytosolic proteome of *H. mustelae *determined by LC-MS Click here for file

Additional file 8 Motifs associated with highly expressed genes in the *H. mustelae *cell envelope proteome Click here for file

Additional file 9 Motifs associated with highly expressed genes in the *H. mustelae *cytosol proteome Click here for file

Additional file 10 **Orthologue comparisons between selected *Campylobacterales*, and *T. maritima *as out-group**. The lower triangle indicates the total number of orthologs in genome-genome comparisons while the upper triangle shows the average sequence identity values expressed in percentages. Click here for file

Additional file 11 Presence in *H. mustelae *of orthologues of *H. pylori *genes identified as essential for colonization of the Mongolian gerbil. Click here for file
