# Supplementary figures and images for: Comparative genomics and proteomics of Helicobacter mustelae, an ulcerogenic and carcinogenic gastric pathogen
Source: BMC Genomics. 2010 Mar 10;11:164. doi: 10.1186/1471-2164-11-164 (PMC2846917; doi:10.1186/1471-2164-11-164)

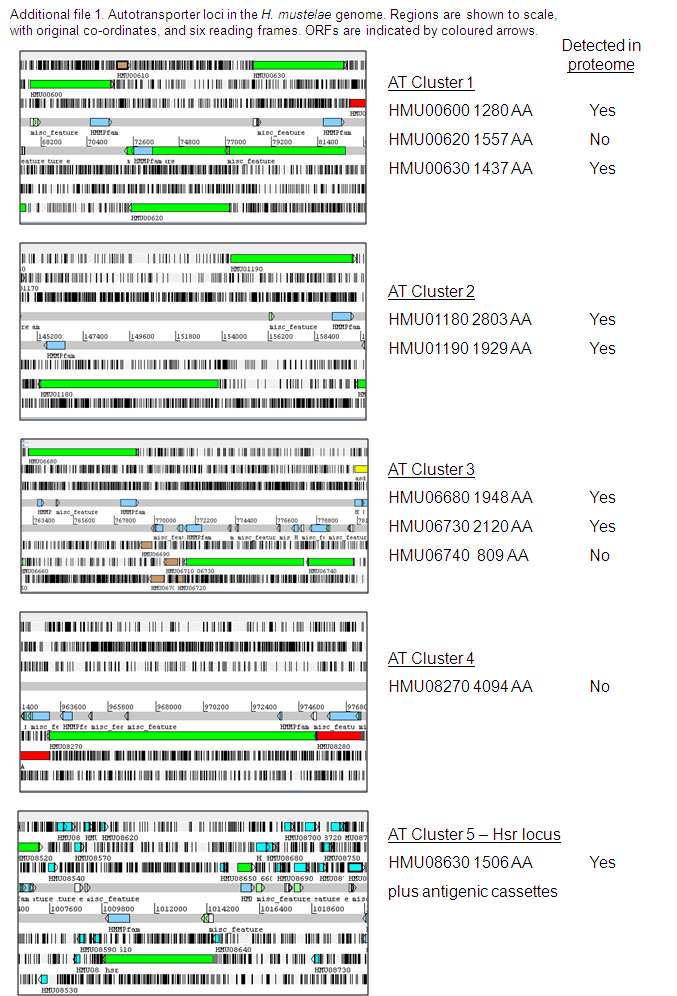

Supplement: Additional file 1 — Autotransporter loci in the H. mustelae genome. [file 1471-2164-11-164-S1.DOCX]
