# Supplementary material for: Comparative genomics and proteomics of Helicobacter mustelae, an ulcerogenic and carcinogenic gastric pathogen
Source: BMC Genomics. 2010 Mar 10;11:164. doi: 10.1186/1471-2164-11-164 (PMC2846917; doi:10.1186/1471-2164-11-164)
Supplement: Additional file 2 — Blood group antigen-associated genes in the genome sequence of H. mustelae, and compared with H. pylori [file 1471-2164-11-164-S2.DOCX]

Additional File 2. Blood group antigen-associated genes in the genome sequence of *H. mustelae*, and compared with *H. pylori*

| ***H. pylori* gene** | **Locus** | **Putative *H. mustelae* ortholog** | **FASTA identity/overlap** | **Predicted protein and function** |
| --- | --- | --- | --- | --- |
| *gluP* | 1174 | HMU0500 | 61% identity in 407 aa | Membrane protein (Transporter) Uptake of glucose/galactose |
| *galE* | 0360 | HMU00090 | 55% identity in 339 aa | UDP-glucose 4-epimerase Synthesis of UDP-galactose |
| *rfbM* | 0043 | HMU13170 | 45% identity in 469 aa | (manA/C)Phosphomannose isomerase Synthesis of GDP-fucose |
| *rfbD* | 0044 | HMU13180 | 65% identity in 378 aa | (gmd) GDP-D-mannose dehydratase Synthesis of GDP-fucose |
| *wbcJ* | 0045 | Not found - HMU00090? |  | Epimerase-reductase? Synthesis of GDP-fucose |
| *rfaJ* | 0159 | HMU10850 | 27% in 211 aa | lipopolysaccharide 1,2-glucosyltransferase |
| *rfaJ* | 0208 | HMU10850 | HP0208 is frameshifted |  |
| *rfaJ* | 1416 | Not found |  |  |
| *galT* | 0826 | Not found |  | b1,4-galactosyltransferase Synthesis of LacNAc |
| *futA* | 0379 | Not found |  | (fucTa) a1,3(4)-fucosyltransferase Synthesis of Lewis x, a, y, b |
| *futB* | 0651 | Not found |  | (fucTb) a1,3(4)-fucosyltransferase Synthesis of Lewis x, a, y, b |
| *futC* | 0094-0093 | HMU12060 | 77% in 280 aa | (fucT2) a1,2-fucosyltransferase Synthesis of H antigens and Lewis y, b |
| *neuA* | 0326 | HMU11730? | 43% in 209 aa | Acylneuraminate cytidyltransferase Synthesis of Sialyl-Lewis x |
| *neuB* | 0178 | HMU11700 | 53% in 333 aa | Sialic acid synthase Synthesis of Sialyl-Lewis x |
| *-* | - | HMU12050 | Not found in Campylobacterales | ABO glycosyltransferase; synthesis of blood groups A, B antigens from H substance |
