# Supplementary material for: Comparative genomics and proteomics of Helicobacter mustelae, an ulcerogenic and carcinogenic gastric pathogen
Source: BMC Genomics. 2010 Mar 10;11:164. doi: 10.1186/1471-2164-11-164 (PMC2846917; doi:10.1186/1471-2164-11-164)
Supplement: Additional file 3 — Motility- and flagellum-associated genes in the genome sequence of H. mustelae, and compared with H. pylori [file 1471-2164-11-164-S3.DOCX]

Additional File 3. Motility- and flagellum-associated genes in the genome sequence of *H. mustelae*, and compared with *H. pylori*

| **Gene** | ***H. mustelae* gene** | ***H. pylori* gene*** | **Protein function/homolog in S. Typhimurium -yes/no (gene designation in S. Typhimurium, if different from *Helicobacter* nomenclature)** | ***H. pylori* promoter if known** |
| --- | --- | --- | --- | --- |
| **Structure and function** | | | | |
| *-* | HMU07160 | HP0114 | flagellin glycosylation/no | ? |
| *flaB* | HMU07150 | HP0115 | flagellin B /no | σ54 |
| *cheZ** | HMU02640 | HP0170 | Remote CheZ orthologue, possibly involved in phosphate flow in the chemotaxis system/yes | ? |
| *fliR* | HMU13230 | HP0173 | regulatory protein of flagellar biogenesis flagellar basal body protein /yes |  |
| *neuB3* | HMU11700 | HP0178 | flagellin glycosylation/no | ? |
| *-* | HMU06070 | HP0232 | secreted protein involved in motility /no | ? |
| *flgI* | HMU03430 | HP0246 | P ring forming protein of basal body /yes | σ70 (?) |
| *flgL* | HMU06870 | HP0295 | HAP-3 homolog /yes | σ54 (?) |
| *flgH* | HMU11740 | HP0325 | L ring forming protein of basal body /yes |  |
| *neuA* | HMU11730 | HP0326 | flagellin glycosylation/no | ? |
| *flmD* | HMU11720 | HP0326b | flagellin glycosylation/no | ? |
| *flaG1* (*flmH*) | HMU11710 | HP0327 | flagella associated protein -glycosylation/no | σ70 (?) |
| *fliF* | HMU11870 | HP0351 | M ring-forming protein of basal body /yes | σ70(?) |
| *fliG* | HMU11860 | HP0352 | flagellar motor switch protein, putative C ring component /yes | σ70 (?) |
| *fliH* | HMU11850 | HP0353 | protein involved in flagellar export apparatus /yes | σ70 (?) |
| *-* | HMU06610 | HP0366 | DegT-like; involved in flagellin glycosylation/no | σ54 and σ28 (?) |
| *hpa2* | --- | HP0410 | paralog of HP hemagglutinin protease - sheath associated/no protein /no | ? |
| *omp11* | HMU01800 | HP0472 | coregulated with late flagellar genes/flagellar sheath protein?/no | σ28 |
| *hpaA3* | --- | HP0492 | Ortholog of HpaA/no | ? |
| *fliN* | HMU04810 | HP0584 | flagellar motor switch protein, putative C ring component /yes | σ70 (?) |
| *flaA* | HMU05840 | HP0601 | Major flagellin A/yes (fliC) | σ28 |
| *fliP'* | HMU02400 | HP0684 | N-terminal domain of basal body protein FliP involved in flagellar export apparatus /yes | ? |
| *fliP''* | HMU02400 | HP0685 | second half of basal body protein FliP† /yes | ? |
| *flaG2* | HMU03850 | HP0751 | gene of unknown function involved in biogenesis of polar flagellum/no | σ70, σ28 (?) |
| *fliD* | HMU03840 | HP0752 | hook-associated protein-2 (HAP-2), filament cap protein FliD/yes | σ70, σ28 (?) |
| *fliS* | HMU03830 | HP0753 | flagellin chaperone protein FliS/yes | σ70, σ28 (?) |
| *fliT* | HMU03820 | HP0754 | Flagellar chaperone | σ70, σ28 (?) |
| *flhB1* | HMU06030 | HP0770 | flagellar basal body protein involved in export /yes | σ70 (?) |
| *hpaA* | --- | HP0797 | flagellar sheath "adhesin" protein -neuraminyllactose binding protein/no | ? |
| *motB* | HMU03570 | HP0815 | flagellar motor protein B/yes | σ70 (?) |
| *motA* | HMU03580 (pseudo) | HP0816 | flagellar motor protein A/yes |  |
| *flaA1* | HMU09920 | HP0840 | flagella-associated protein involved in glycosylation/no | ? |
| *flgE1* | HMU00850 | HP0870 | flagellar hook protein /yes | σ54 |
| *fliK* | HMU07800 (pseudo) | HP0906 | flagellar substrate switch protein | σ70 |
| *flgD* | HMU07810 | HP0907 | flagellar hook scaffolding protein /yes | σ54 |
| *flgE2*(*flgF*) | HMU07820 | HP0908 | putative flagellar hook basal body protein homolog /no | σ54 |
| *-* | HMU13450 | HP0958 | stabilizes flagellar σ54 factor/no | ? |
| *fliY* | HMU12760 | HP1030 | FliY protein /no | σ70, σ28 (?) |
| *fliM* | HMU12770 | HP1031 | flagellar motor switch protein putative C ring component /yes | σ70, σ28 (?) |
| *flhG/ylxH* | HMU12800 | HP1034 | cooperates with FlhF in polar flagellar biosynthesis/no | σ70, σ28 (?) |
| *flhF* | HMU12810 | HP1035 | flagellar biosynthesis protein of unknown function /no | σ70, σ28 (?) |
| *flhA* | HMU05260 | HP1041 | flagellar basal body protein involved in export /yes | ? |
| *-* | HMU01740 | HP1051 | hypothetical protein | σ28 |
|  | HMU01730 | HP1052 | deacetylase EnvA/LpxC (cell envelope biosynthesis) | σ28 |
| *-* | HMU03600 | HP1076 | hypothetical protein coregulated with middle flagellar genes | σ54 |
| *flgG1* | HMU11810 | HP1092 | Homologous to distal rod protein of flagellar basal body, putative functional FlgF substitute /no-yes or rod-hook junction protein | σ54 |
| *FlgK* | HMU04500 | HP1119 | hook-associated protein 1 (HAP 1) /yes | σ54 |
| *-* | HMU04510 | HP1120 | coregulated with flgK | σ54 |
| *-* | HMU01930 | HP1154 | hypothetical protein | σ54 |
| *murG* | HMU04670? | HP1155 | peptidoglycan biosynthesis | σ54 |
| *-* | --- | HP1192 | secreted protein involved in motility /no | ? |
| *flgJ*?* | HMU02380 | HP1233 | remote orthologue to flagellar muraminidase /yes | σ54 |
| *FliQ* | HMU05780 | HP1419 | flagellar basal body protein involved in export /yes | ? |
| *FliI* | HMU10250 | HP1420 | flagella-associated ATPase, energizer of flagellar export /yes | ? |
| *-* | HMU08440 | HP1462 | secreted protein involved in motility /no | ? |
| *fliE* | HMU12450 | HP1557 | flagellar basal body protein /yes | σ70 (?) |
| *flgC* | HMU12440 | HP1558 | proximal rod protein of basal body /yes | σ54 |
| *flgB* | HMU12430 | HP1559 | proximal rod protein of basal body /yes | σ54 |
| *flhB2* | HMU11590 | HP1575 | homolog of FlhB protein basal body /no | ? |
| *flgG2* | HMU01910 | HP1585 | distal rod protein of flagellar basal body /yes | σ70-like/σ54 |
| **Chemotaxis** | | | | |
| *cheV1* | ? | HP0019 | CheW/CheY hybrid chemotaxis protein /no | ? |
| *tlp* | HMU05230 | -- | Chemotaxis receptor/specificity unknown most similar to t*lpB*/HP0103 |  |
| *tlpC* | --- | HP0082 | receptor protein of chemotaxis signalling system /specificity unknown | ? |
| *tlpA* | --- | HP0099 | chemotaxis receptor /specificity unknown | ? |
| *tlpB* | HMU05230 | HP0103 | chemotaxis receptor /specificity unknown | ? |
| *cheZ** | HMU02640 | HP0170 | Remote CheZ orthologue, possibly involved in phosphate flow in the chemotaxis system /yes | ? |
| *cheW* | HMU03760 | HP0391 | Chemotaxis adaptor protein /yes |  |
| *cheA* | HMU03770 | HP0392 | receptor coupled histidine kinase /yes, but fused to CheY2 domain in HP | ? |
| *cheV3* | HMU06240 | HP0393 | hybrid CheW/CheY chemotaxis protein /no | ? |
| *tlpD* | HMU11680 (?) | HP0599 | soluble chemotaxis receptor /specificity unknown | ? |
| *tlpPAS* | HMU5990 | -- | chemotaxis receptor /specificity unknown |  |
| *cheV2* | HMU14170 | HP0616 | hybrid CheW/CheY chemotaxis protein | ? |
| *cheY1* | HMU02170 | HP1067 | chemotaxis effector protein - link to basal body motor /yes | σ70 |
| **Regulation** | | | | |
| *flgS* | HMU03450 | HP0244 | histidine kinase involved in activation of FlgR and RpoN/no | σ 70 |
| *flgR* | HMU08790 | HP0703 | NtrC homolog; transcriptional activator of σ54-regulated motility genes (response regulator) /no |  |
| *rpoN* | HMU10620 | HP0714 | σ54 subunit of DNA-dependent RNA polymerase | ? |
| *-* | HMU13450 | HP0958 | RpoN-chaperone; *flaA* mRNA chaperone/no | ? |
| *fliA* | HMU12780 | HP1032 | σ28 subunit of DNA-dependent RNA polymerase /"flagellar" σ factor /yes | σ70, σ28 (?) |
| *flgM* | HMU04480 | HP1122 | Flagellar anti-σ28 factor | σ70, σ54, σ28 |

**H. pylori*  strain 26695 locus numbers are referred to

†HP0684 and HP0685 are one continuous ORF in strains J99 and HPAG1.
