# Supplementary material for: Comparative genomics and proteomics of Helicobacter mustelae, an ulcerogenic and carcinogenic gastric pathogen
Source: BMC Genomics. 2010 Mar 10;11:164. doi: 10.1186/1471-2164-11-164 (PMC2846917; doi:10.1186/1471-2164-11-164)
Supplement: Additional file 4 — Protein secretion-associated genes in the genome sequence of H. mustelae, and compared with H. pylori [file 1471-2164-11-164-S4.DOCX]

Additional file 4. Protein secretion-associated genes in the genome sequence of *H. mustelae*, and compared with *H. pylori*

| **Gene** | ***H. mustelae* gene** | ***H. pylori* gene** | **Comment** |
| --- | --- | --- | --- |
| **Sec system** | | | |
| SecA | HMU10950 | HP0786 | *H. pylori* HP0539 (cagL, cag18) also reported as SecA homologue[140] |
| SecB |  | - | Component not always required in *E. coli* |
| SecD | HMU10070 | HP1550 |  |
| SecE | HMU05235 | HP1203-1204 interval | Difficult to annotate, not in original *H. pylori* annotations, but located by Médigue *et al.* [141] |
| SecF | HMU10060 | HP1549 |  |
| SecG | HMU09610 | HP1255 |  |
| SecY | HMU10360 | HP1300 | H. pylori HP0535 (CagR) also reported as SecY homologue[140] |
| YajC | HMU10080 | HP1551 | Sec |
| YidC | HMU09470 | HP1450 | Previously designated OxaA |
| SRP (ffh) | HMU03400 | HP1152 |  |
| FtsY | HMU13280 | HP0763 |  |
| Trigger factor (tig) | HMU10240 | HP0795 |  |
| **Tat system** |  |  |  |
| TatA | HMU02290 | HP0320? | Present in *H. hepaticus* (HH_0708) |
| TatB | HMU01100 | HPAG1_0387 | Annotated in *H. pylori*  HPAG1[142]; and *H. hepaticus* HH_1489 |
| TatC | HMU01110 | HP1061 |  |
| TatD | HMU11570 | HP1573 |  |
| **Type II General Secretory Pathway** | | | |
| GspC | Not found | - |  |
| GspD | HMU14330 (pseudo) | - | OM pore; *H. hepaticus* potential PilQ homolog HH_1115 |
| GspE | HMU14310 | ? | ATP binding; homologues in *H. pylori* are Vir-type ATPases HP1421 (VirB11) and HP0525 (cag). Potential *H. hepaticus* PilT homologue HH_1116 |
| GspF | HMU14300 | - | Potential *H. hepaticus* PilG homologue HH_1117 |
| GspG | HMU14240 | - | Pseudopilin |
| GspO | HMU05350 | - | Prepilin peptidase homolog, but only over limited stretch of the protein |
| HopD | Not found | - | Type IV pilin peptidase. Present in *H. hepaticus* (HH_0603) |
| PilT | HMU11310 |  | ATPase, similar to CtsE |
| **Other factors** | | | |
| LspA | HMU01410 | HP0074 | Lipoprotein signal peptidase (signal peptidase II) |
| LepB | HMU06460 | HP0576 | Classical signal peptidase I; also called LepP |
| YaeT (Omp85) | HMU02870 | HP0655 | Essential outer membrane protein, helps autotransporter secretion. Annotated as protective surface antigen D15 in *H. pylori* 26695 |

Some genes were not annotated in *H. pylori* 26695, in which case the HPAG1 or J99 gene numbers are cited.

- indicates not annotated
