# Supplementary material for: Comparative genomics and proteomics of Helicobacter mustelae, an ulcerogenic and carcinogenic gastric pathogen
Source: BMC Genomics. 2010 Mar 10;11:164. doi: 10.1186/1471-2164-11-164 (PMC2846917; doi:10.1186/1471-2164-11-164)
Supplement: Additional file 5 — Presence of homopolyeric tracts within/between H. mustelae genes, and homopolymer length variation in sequence data [file 1471-2164-11-164-S5.DOCX]

Additional file 5. Presence of homopolyeric tracts within/between *H. mustelae* genes, and homopolymer length variation in sequence data

| **Motif** | **Gene affected** | **Co-ordinate^a^** | **Identifying sequence** | **Predicted function of affected gene** | **Number of reads x motif in shotgun data** |
| --- | --- | --- | --- | --- | --- |
| **Intragenic homopolymers** | | | | | |
| G11 | HMU02610 | 313337 | GAGTTGCGGGGGGGGGGGCACTAAACACTCTAGTCGCTCTA | putative signaling protein; extreme C-term only affected | 10 x G11 |
| G9 | HMU03040 | 364010 | TTGAGGTAGCACTTTTTGCCAAGGGGGGGGGTTGAGTTTTTGTGAGGGCCTGATTC | putative galactosyltransferase - homolog of Campy LOS gene | 10 x G9 |
| C9 | HMU04930 | 566286 | GGCACTTGTGCGGCACTAGCACCCCCCCCCGGGCCACTGCCACTTTCGT | putative OMP | 10 x C9 |
| C9 | HMU05300 | 614467 | ACAAAGATGATATACTCCCCCCCCCTTTGCTTTTGCTGCAGTGAGAGA | LPS biosynthesis pseudogene | 10 x C9 |
| C9 | HMU05320 | 616657 | ACAAAGATGATATACTCCCCCCCCCCTTTGCTTTTGCTGCAGTGAGAG | putative LPS biosynthesis-related glycosyltransferase | 10 x C9 |
| C10 | HMU06550 | 749167 | CTATCATCTCGCCCCCCCCCCATTATTTTTGTATACATCAAAAG | putative glycosyltransferase | 12 x C10 |
| G12 | HMU06700 | 769978 | GCCAGAGTGCTTTTTTGCCCGCGGGGGGGGGGGCTAAAAAGTGTCTA | putative glycosyltransferase, partial gene (pseudogene) | 1 x G12, 4 x G11, 2 x G10 |
| G10 | HMU06720 | 771287 | TGGATTTCCCTGTAGACTTGGGGGGGGGGTGGTAGAATGGTGGGTGAT | putative glycosyltransferase, partial gene (pseudogene) | 10 x G10 |
| C9 | HMU09730 | 1106553 | GCTTGCTAGCTTGAAAGGGGGGGGGCACCATAAAAAAGGGGGAGA | putative glycosyl transferase domain protein | 12 x G9 |
| G9 | HMU10770 | 1198504 | AGTTGATAAAGGGGAGGAACCCCCCCCCACTGCTTAGAGTGATTTGCT | putative beta-1,4-N-acetylgalactosaminyltransferase; (Campy CMP-Neu5Ac synthase) | 4 x G9 |
| C9 | HMU10850 | 1206064 | TATAGCATTACAAATAATACCCCCCCCCTACGGTTTATACAT | putative lipopolysaccharide 1,3-galactosyltransferase; extreme C-term only affected | 10 x G9 |
| G9 | HMU11990 | 1319521 | AGCGAAGAGATAGCAATTATTGGGGGGGGGCAATATATAGTCTTTGCGC | putative type IIS restriction /modification enzyme, N-terminal half | 7 x G9 |
| GA8 | HMU11990 | 1320163 | GAATCAGAATTCAGCAGCAAGAGAGAGAGAGAGAGATCTGAAATAATTGATAAAGTC | putative type IIS restriction /modification enzyme, N-terminal half | 5 x (GA)8, 3 x (GA)9, 1 x (GA)7 |
| **Intergenic homopolymers** | | | | | |
| Morif | Homopolymer co-ordinates | **Co-ordinate^a^** | **Identifying sequence** | **Predicted function of affected gene** | **Number of reads x motif in shotgun data** |
| G9 | 22390..22400 | 22372 | TTGCGCGAAGTTTACTGTCCCCCCCCCCCTAAGAGCAATCCGCTTAA | Upstream predicted OMP HMU00180 | 6 x G11, 1x G10, 2 x G12 |
| G14 | 153965..153978 | 153948 | ATTTGTAGGAGTTTTGCGGGGGGGGGGGGGGTGCATTTTGCTGCAT | Intergenic two divergently transcribed autotransporter genes | 9 x G14, 1 x G12 |
| G11 | 617013..617023 | 616994 | GAGTTTTAGTATGGTTTGCGGGGGGGGGGGTGATTTTGGTGCGTATTCATGGGGTTT | 85 nt upstream HMU05320 | 2 x G11, 1x G12, 1 x G13 |
| C9 | 667921..667929 | 667901 | GATAAAATTTCTTTGATTTACCCCCCCCCTATATTTACCCCCCCCTATATTT | Upstream *fliQ* | 5 x C9 |
| C9 | 728187..728195 | 728167 | CTGAAGAGAGTTTTCTCCTGCCCCCCCCCTGGCAGCTTTTTTCTCA | Upstream HMU06320 Putative hypothetical protein | 4 x C9 |
| C10 | 1074178..1074187 | 1074158 | GATTGAGAAAAAAGCTGCCAGGGGGGGGGGCAGGAGAAAACTCTCTTCAG | Upstream HMU09330 putative ADH/glycosyltransferase | 6 x C10 |
| C11 | 1075397..1075407 | 1075379 | TTGAGAAAAAAGCTGCCAGGGGGGGGGGGCAGGAGAAAACTCTCTTAAGACAAT | Upstream HMU09340 putative glycosyltransferase | 5 x C11 |
| C12 | 1325896..1325907 | 1325878 | CTTAAAAAAGCACTAAATCCCCCCCCCCCCGCTAAAAGCAAGCATTT | Upstream putative alpha-1,2-fucosyltransferase | 6 x C12 |

a. Co-ordinate for start of the identifying sequence
