# Supplementary material for: Comparative genomics and proteomics of Helicobacter mustelae, an ulcerogenic and carcinogenic gastric pathogen
Source: BMC Genomics. 2010 Mar 10;11:164. doi: 10.1186/1471-2164-11-164 (PMC2846917; doi:10.1186/1471-2164-11-164)
Supplement: Additional file 6 — The envelope proteome of H. mustelae determined by LC-MS. [file 1471-2164-11-164-S6.DOCX]

Additional file 6. The envelope proteome of *H. mustelae* determined by LC-MS.

| **Rank^a^** | **Locus** | **Annotation** | **Mw Da** | **MOWSE Score^b^** | **% Coverage^c^** | **emPAI^d^** | **Mol%** |
| --- | --- | --- | --- | --- | --- | --- | --- |
| 1 | HMU14370 | cft ferritin | 19,056 | 455 | 53 | 2.73 | 5.74 |
| 2 | HMU03320 | ahpC alkyl hydroperoxide reductase | 22,088 | 806 | 68 | 2.48 | 5.22 |
| 3 | HMU12840 | aroQ 3-dehydroquinate dehydratase | 17,477 | 105 | 21 | 2.16 | 4.55 |
| 4 | HMU14250 | undefined product; COG4969, tfp pilus assembly protein, major pilin PilA [cell motility and secretion / Intracellular trafficking and secretion] | 15,880 | 241 | 50 | 1.42 | 3 |
| 5 | HMU00320 | undefined product; no putative conserved domains detected, hypothetical protein Lreu23DRAFT_1924 [L. reuteri 100-23]: 35% ID | 8,942 | 158 | 33 | 1.37 | 2.89 |
| 6 | HMU14210 | fldA flavodoxin 1 | 18,147 | 231 | 25 | 1.31 | 2.76 |
| 7 | HMU03050 | ureA fusion of urease beta and gamma subunits | 25,199 | 683 | 60 | 1.15 | 2.43 |
| 8 | HMU03060 | ureB urease alpha subunit | 61,146 | 1,218 | 37 | 1.05 | 2.21 |
| 9 | HMU04000 | groEL 60 kD chaperonin (cpn60) | 57,443 | 1,714 | 51 | 1.04 | 2.18 |
| 10 | HMU12690 | possible bacterioferritin | 17,198 | 380 | 32 | 1.02 | 2.14 |
| 11 | HMU03500 | putative amino-acid transporter periplasmic solute-binding protein | 30,533 | 626 | 52 | 0.96 | 2.02 |
| 12 | HMU01210 | tpx probable thiol peroxidase | 17,993 | 350 | 38 | 0.83 | 1.75 |
| 13 | HMU00630 | putative putative autotransporter protein | 151,727 | 1,371 | 25 | 0.82 | 1.73 |
| 14 | HMU13930 | putative putative exported protein; COG1464, NlpA, ABC-type metal ion transport system, periplasmic component/surface antigen [inorganic ion transport and metabolism] | 28,862 | 425 | 30 | 0.82 | 1.73 |
| 15 | HMU01180 | putative putative membrane-anchored cell surface protein | 281,808 | 2,432 | 22 | 0.82 | 1.72 |
| 16 | HMU03120 | tuf elongation factor TU | 43,570 | 850 | 48 | 0.78 | 1.64 |
| 17 | HMU04030 | petA putative putative ubiquinol-cytochrome C reductase iron-sulfur subunit | 17,916 | 227 | 46 | 0.78 | 1.64 |
| 18 | HMU06410 | undefined product; No putative conserved domains detected, hypothetical protein Hac_1480 [Helicobacter acinonychis str. Sheeba]: 29% ID | 20,229 | 254 | 18 | 0.78 | 1.64 |
| 19 | HMU03990 | groES 10 kD chaperonin (cpn10) | 10,185 | 157 | 31 | 0.72 | 1.51 |
| 20 | HMU09770 | trxA thioredoxin | 11,553 | 194 | 28 | 0.72 | 1.51 |
| 21 | HMU08630 | putative putative outer membrane autotransporter | 155,228 | 968 | 14 | 0.65 | 1.37 |
| 22 | HMU05030 | putative putative hydantoinase A | 78,444 | 785 | 30 | 0.55 | 1.15 |
| 23 | HMU13940 | putative putative exported protein; COG1464: ABC-type metal ion transport system, periplasmic component/surface antigen [inorganic ion transport and metabolism] | 29,374 | 430 | 29 | 0.55 | 1.15 |
| 24 | HMU05020 | undefined product; COG4647/pfam08882, acetone_carb_G | 14,279 | 213 | 29 | 0.49 | 1.04 |
| 25 | HMU01190 | putative hypothetical glycine-rich autotransporter protein | 194,616 | 1,281 | 19 | 0.48 | 1.02 |
| 26 | HMU00600 | putative LPXTG surface protein | 133,006 | 651 | 14 | 0.48 | 1 |
| 27 | HMU09610 | secG putative putative protein-export membrane protein | 12,734 | 81 | 21 | 0.47 | 0.98 |
| 28 | HMU12860 | sodB superoxide dismutase (Fe) | 24,576 | 231 | 23 | 0.47 | 0.98 |
| 29 | HMU14090 | putative thioredoxin | 11,796 | 138 | 30 | 0.47 | 0.98 |
| 30 | HMU11370 | undefined product; no putative conserved domains detected, hypothetical protein Abu_2077 [Arcobacter butzleri RM4018]: 37% | 7,677 | 92 | 26 | 0.47 | 0.98 |
| 31 | HMU05010 | putative hydantoin hydantoinase A | 70,769 | 835 | 31 | 0.46 | 0.96 |
| 32 | HMU10410 | rpsH 30S ribosomal protein S8 | 14,807 | 247 | 32 | 0.43 | 0.91 |
| 33 | HMU10080 | putative putative membrane protein | 10,902 | 103 | 17 | 0.43 | 0.89 |
| 34 | HMU04360 | atpC ATP synthase F1 sector epsilon subunit | 13,211 | 108 | 16 | 0.43 | 0.89 |
| 35 | HMU04350 | atpD ATP synthase F1 sector beta subunit | 51,310 | 581 | 23 | 0.42 | 0.89 |
| 36 | HMU10380 | rpsE 30S ribosomal protein S5 | 15,592 | 199 | 33 | 0.43 | 0.89 |
| 37 | HMU09160 | undefined product; no putative conserved hits detected, hypothetical protein HH1743 [Helicobacter hepaticus ATCC 51449]: 32% ID | 43,936 | 335 | 14 | 0.40 | 0.85 |
| 38 | HMU10290 | rpoA DNA-directed RNA polymerase alpha chain | 37,551 | 320 | 24 | 0.39 | 0.82 |
| 39 | HMU05040 | putative hydantoin utilization protein B | 83,272 | 514 | 23 | 0.38 | 0.8 |
| 40 | HMU05840 | putative flagellin | 53,982 | 501 | 27 | 0.37 | 0.79 |
| 41 | HMU10120 | peb4[1]f2 major antigenic peptide PEB3ll binding factor 2 | 31,816 | 371 | 32 | 0.36 | 0.76 |
| 42 | HMU04050 | petC putative putative ubiquinol-cytochrome C reductase cytochrome C subunit | 33,425 | 299 | 25 | 0.35 | 0.74 |
| 43 | HMU04380 | exbD3 exbD olR family transport protein | 14,662 | 132 | 23 | 0.35 | 0.74 |
| 44 | HMU06080 | hypothetical protein Cj0372; COG0754/pfarm03738: Glutathionylspermidine synthase [amino acid transport and metabolism] | 44,843 | 197 | 12 | 0.35 | 0.74 |
| 45 | HMU11040 | fliL possible flagellar protein | 18,741 | 175 | 18 | 0.33 | 0.7 |
| 46 | HMU04330 | atpA ATP synthase F1 sector alpha subunit | 55,012 | 371 | 20 | 0.32 | 0.67 |
| 47 | HMU07380 | hupB DNA-binding protein HU homolog | 10,122 | 142 | 26 | 0.29 | 0.61 |
| 48 | HMU03350 | rplS 50S ribosomal protein L19 | 13,546 | 226 | 28 | 0.29 | 0.61 |
| 49 | HMU03180 | rplL 50S ribosomal protein L7 /L12 | 12,946 | 97 | 18 | 0.27 | 0.58 |
| 50 | HMU01920 | htrA serine protease (protease DO) | 45,914 | 305 | 17 | 0.27 | 0.58 |
| 51 | HMU13670 | undefined product; no putative conserved domains detected, hypothetical protein HH0645 [Helicobacter hepaticus ATCC 51449]: 23% ID | 19,128 | 153 | 16 | 0.27 | 0.58 |
| 52 | HMU13050 | putative putative transmembrane protein | 39,881 | 240 | 23 | 0.26 | 0.54 |
| 53 | HMU02920 | exbD2 putative putative exbD/tolR family transport protein | 14,170 | 142 | 12 | 0.26 | 0.54 |
| 54 | HMU09500 | putative putative exbD/tolR family transport protein | 14,965 | 135 | 21 | 0.26 | 0.54 |
| 55 | HMU13900 | frdB fumarate reductase iron-sulfur protein | 27,821 | 213 | 19 | 0.24 | 0.51 |
| 56 | HMU02930 | exbB2 putative putative exbB/tolQ family transport protein | 16,053 | 76 | 8 | 0.23 | 0.49 |
| 57 | HMU05270 | rpsO 30S ribosomal protein S15 | 10,411 | 181 | 37 | 0.23 | 0.49 |
| 58 | HMU10450 | rplN 50S ribosomal protein L14 | 13,373 | 188 | 26 | 0.22 | 0.46 |
| 59 | HMU10960 | katA catalase | 52,490 | 321 | 21 | 0.22 | 0.46 |
| 60 | HMU05800 | undefined product; no putative conserved hits detected, hypothetical protein HH0147 [Helicobacter hepaticus ATCC 51449]: 36% ID | 62,920 | 333 | 9 | 0.22 | 0.46 |
| 61 | HMU10430 | rplE 50S ribosomal protein L5 | 20,258 | 113 | 12 | 0.21 | 0.45 |
| 62 | HMU10630 | putative putative periplasmic protein | 21,100 | 228 | 18 | 0.21 | 0.43 |
| 63 | HMU04120 | putative putative outer membrane component of efflux system | 50,431 | 242 | 12 | 0.19 | 0.4 |
| 64 | HMU05990 | putative putative MCP-type signal transduction protein | 47,627 | 297 | 15 | 0.19 | 0.4 |
| 65 | HMU02630 | putative putative protease; | 49,578 | 83 | 14 | 0.19 | 0.39 |
| 66 | HMU02170 | cheY chemotaxis regulatory protein | 13,894 | 55 | 9 | 0.18 | 0.38 |
| 67 | HMU03460 | gdh NADP-specific glutamate dehydrogenase | 49,935 | 270 | 14 | 0.18 | 0.38 |
| 68 | HMU10560 | rpsJ 30S ribosomal protein S10 | 11,807 | 102 | 31 | 0.18 | 0.38 |
| 69 | HMU11960 | undefined product; pfam01569, PAP2 superfamily. | 22,230 | 58 | 8 | 0.18 | 0.38 |
| 70 | HMU05390 | rpsI 30S ribosomal protein S9 | 14,313 | 126 | 20 | 0.17 | 0.36 |
| 71 | HMU12190 | undefined product; no putative conserved domains detected, hypothetical protein HH1863 [H. hepaticus ATCC 51449]: 35% ID | 19,382 | 134 | 17 | 0.17 | 0.36 |
| 72 | HMU05420 | putative putative pyruvate-flavodoxin oxidoreductase | 44,431 | 113 | 8 | 0.17 | 0.35 |
| 73 | HMU01070 | hypothetical protein Cj0449c; COG2841, uncharacterized protein conserved in bacteria [Function unknown]. | 8,772 | 55 | 14 | 0.17 | 0.35 |
| 74 | HMU00920 | HIT-family protein | 12,412 | 70 | 9 | 0.15 | 0.33 |
| 75 | HMU10320 | rpsM 30S ribosomal protein S13 76 | 13,576 | 99 | 22 | 0.15 | 0.33 |
| 76 | HMU05650 | undefined product; pfam02521, HP_OMP_2, Putative outer membrane protein | 51,181 | 115 | 6 | 0.16 | 0.33 |
| 77 | HMU10470 | undefined product COG0255/pfam00831, ribosomal protein L29 [translation, ribosomal structure and biogenesis] | 5,941 | 70 | 26 | 0.15 | 0.33 |
| 78 | HMU09540 | csrA carbon storage regulator homolog | 8,394 | 61 | 17 | 0.15 | 0.31 |
| 79 | HMU04140 | putative putative integral membrane component of efflux system | 117,261 | 262 | 9 | 0.14 | 0.3 |
| 80 | HMU05640 | undefined product; pfam02521: HP_OMP_2, putative outer membrane protein. | 51,804 | 80 | 6 | 0.14 | 0.29 |
| 81 | HMU03190 | putative DNA-directed RNA polymerase beta' chain | 323,245 | 1,237 | 15 | 0.13 | 0.28 |
| 82 | HMU10540 | rplD 50S ribosomal protein L4 | 22,908 | 92 | 17 | 0.13 | 0.27 |
| 83 | HMU10280 | rplQ 50S ribosomal protein L17 | 13,224 | 63 | 15 | 0.13 | 0.27 |
| 84 | HMU02340 | accB putative putative biotin carboxyl carrier protein of acetyl-CoA carboxylase | 16,525 | 57 | 7 | 0.12 | 0.26 |
| 85 | HMU10490 | rpsC 30S ribosomal protein S3 | 25,408 | 147 | 19 | 0.12 | 0.26 |
| 86 | HMU11240 | undefined product; no putative conserved domains detected, hypothetical protein Hac_0738 [H. acinonychis str. Sheeba]: 37% | 14,855 | 61 | 10 | 0.12 | 0.26 |
| 87 | HMU06730 | putative putative outer membrane autotransporter | 228,457 | 203 | 5 | 0.12 | 0.25 |
| 88 | HMU01010 | putative Major Facilitator Superfamily protein | 37,592 | 42 | 2 | 0.12 | 0.24 |
| 89 | HMU03770 | cheA chemotaxis histidine kinase | 85,293 | 203 | 10 | 0.11 | 0.24 |
| 90 | HMU13600 | undefined product; COG3009, uncharacterized protein conserved in bacteria [Function unknown] | 21,548 | 76 | 10 | 0.12 | 0.24 |
| 91 | HMU10510 | rpsS 30S ribosomal protein S19 | 10,600 | 70 | 12 | 0.11 | 0.23 |
| 92 | HMU14190 | undefined product; no putative conserved domains detected, unknown; OrfL [H. pylori]: 28% ID | 24,257 | 40 | 6 | 0.11 | 0.23 |
| 93 | HMU04130 | putative putative membrane fusion component of efflux system | 25,852 | 122 | 11 | 0.11 | 0.22 |
| 94 | HMU07390 | ndk nucleoside diphosphate kinase | 15,293 | 43 | 6 | 0.10 | 0.21 |
| 95 | HMU04040 | petB putative putative ubiquinol-cytochrome C reductase cytochrome B subunit | 48,215 | 81 | 3 | 0.10 | 0.21 |
| 96 | HMU06680 | putative hypothetical glycine-rich autotransporter protein | 210,555 | 94 | 3 | 0.09 | 0.2 |
| 97 | HMU02150 | putA putative putative proline dehydrogenase/delta-1-pyrroline-5-carboxylate dehydrogenase | 133,560 | 139 | 6 | 0.09 | 0.2 |
| 98 | HMU04300 | undefined product; COG0711, AtpF, F0F1-type ATP synthase, subunit b [energy production and conversion]. | 15,613 | 67 | 8 | 0.10 | 0.2 |
| 99 | HMU04410 | pal peptidoglycan associated lipoprotein (omp18) | 18,949 | 74 | 8 | 0.09 | 0.19 |
| 100 | HMU03140 | nusG putative putative transcription antitermination protein | 19,998 | 60 | 7 | 0.09 | 0.19 |
| 101 | HMU10530 | rplW 50S ribosomal protein L23 | 10,531 | 79 | 15 | 0.09 | 0.19 |
| 102 | HMU06890 | rplU 50S ribosomal protein L21 | 11,677 | 70 | 10 | 0.09 | 0.19 |
| 103 | HMU06490 | putative putative membrane protein | 13,741 | 57 | 14 | 0.09 | 0.18 |
| 104 | HMU04240 | putative putative outer membrane autotransporter | 185,366 | 227 | 2 | 0.08 | 0.18 |
| 105 | HMU12200 | putative putative lipoprotein | 22,531 | 41 | 6 | 0.09 | 0.18 |
| 106 | HMU11970 | rpsU 30S ribosomal protein S21 | 8,656 | 47 | 13 | 0.09 | 0.18 |
| 107 | HMU05580 | pspA protease | 31,971 | 65 | 4 | 0.08 | 0.17 |
| 108 | HMU06070 | putative putative flagellar motility protein | 20,857 | 58 | 7 | 0.08 | 0.17 |
| 109 | HMU10300 | rpsD 30S ribosomal protein S4 | 24,016 | 79 | 11 | 0.08 | 0.17 |
| 110 | HMU10400 | rplF 50S ribosomal protein L6 | 19,675 | 70 | 8 | 0.08 | 0.17 |
| 111 | HMU08030 | hypothetical protein Cj0073c; COG1556, uncharacterized conserved protein [function unknown] | 23,595 | 39 | 4 | 0.08 | 0.17 |
| 112 | HMU04320 | atpH ATP synthase F1 sector delta subunit | 20,999 | 67 | 7 | 0.07 | 0.16 |
| 113 | HMU05430 | putative pyruvate-flavodoxin oxidoreductase | 35,085 | 77 | 5 | 0.08 | 0.16 |
| 114 | HMU09370 | putative putative secreted protease; COG0793, Periplasmic protease [cell envelope biogenesis, outer membrane] | 54,758 | 156 | 9 | 0.07 | 0.16 |
| 115 | HMU04310 | atpF ATP synthase F0 sector B subunit | 19,995 | 42 | 6 | 0.07 | 0.15 |
| 116 | HMU12520 | rpsR 30S ribosomal protein S18 | 10,360 | 45 | 10 | 0.07 | 0.15 |
| 117 | HMU07710 | putative ankyrin-repeat containing protein | 23,116 | 47 | 10 | 0.07 | 0.14 |
| 118 | HMU01620 | kdsA 2-dehydro-3-deoxyphosphooctonate aldolase | 29,464 | 44 | 4 | 0.07 | 0.14 |
| 119 | HMU10110 | putative putative fructose-1,6-bisphosphate aldolase | 33,782 | 44 | 4 | 0.07 | 0.14 |
| 120 | HMU03200 | rpsL 30S ribosomal protein S12 | 14,215 | 46 | 8 | 0.06 | 0.14 |
| 121 | HMU11210 | undefined product; pfam05036: SPOR sporulation related domain involved in binding peptidoglycan. | 28,128 | 52 | 5 | 0.07 | 0.14 |
| 122 | HMU02430 | hypothetical protein Cj0459c | 9,393 | 78 | 19 | 0.06 | 0.13 |
| 123 | HMU07210 | putative putative oxidoreductase | 50,905 | 109 | 8 | 0.06 | 0.13 |
| 124 | HMU10060 | secF protein-export membrane protein | 36,634 | 48 | 3 | 0.06 | 0.12 |
| 125 | HMU02140 | putP sodium/proline symporter | 64,546 | 72 | 2 | 0.06 | 0.12 |
| 126 | HMU05230 | putative MCP-domain signal transduction protein | 62,184 | 175 | 6 | 0.06 | 0.12 |
| 127 | HMU10370 | rplO 50S ribosomal protein L15 | 14,566 | 41 | 10 | 0.06 | 0.12 |
| 128 | HMU11790 | ilvC ketol-acid reductoisomerase | 36,381 | 40 | 2 | 0.06 | 0.12 |
| 129 | HMU11070 | nifU protein homolog | 35,917 | 51 | 5 | 0.06 | 0.12 |
| 130 | HMU00470 | mreB homolog of E. coli rod shape-determining protein | 36,913 | 63 | 4 | 0.05 | 0.11 |
| 131 | HMU01260 | ald alanine dehydrogenase | 39,844 | 39 | 3 | 0.05 | 0.11 |
| 132 | HMU04740 | oorA OORA subunit of 2-oxoglutarate:acceptor oxidoreductase | 40,773 | 56 | 5 | 0.05 | 0.11 |
| 133 | HMU08830 | undefined product; no putative conserved domains detected, hypothetical protein jhp0639 [H. pylori J99]: 31% | 36,273 | 113 | 6 | 0.05 | 0.11 |
| 134 | HMU07150 | flaB flagellin | 54,011 | 172 | 7 | 0.09 | 0.1 |
| 135 | HMU10550 | rplC 50S ribosomal protein L3 | 21,082 | 94 | 8 | 0.05 | 0.1 |
| 136 | HMU03160 | rplA 50S ribosomal protein L1 | 25,256 | 56 | 6 | 0.05 | 0.1 |
| 137 | HMU00170 | putative putative hemolysin | 226,962 | 92 | 2 | 0.04 | 0.09 |
| 138 | HMU12770 | fliM flagellar motor switch protein | 40,063 | 46 | 4 | 0.05 | 0.09 |
| 139 | HMU00850 | flgE2 flagellar hook subunit protein | 75,460 | 40 | 1 | 0.04 | 0.09 |
| 140 | HMU00370 | hypothetical protein Cj1710c | 75,922 | 92 | 5 | 0.05 | 0.09 |
| 141 | HMU14040 | putative putative zinc protease | 49,968 | 76 | 3 | 0.04 | 0.09 |
| 142 | HMU00950 | rpsA 30S ribosomal protein S1 | 60,196 | 127 | 5 | 0.04 | 0.09 |
| 143 | HMU13890 | frdA fumarate reductase flavoprotein subunit | 72,752 | 127 | 4 | 0.04 | 0.09 |
| 144 | HMU01370 | undefined product; PRK10769, folA, dihydrofolate reductase. | 40,098 | 62 | 5 | 0.04 | 0.09 |
| 145 | HMU04340 | atpG ATP synthase F1 sector gamma subunit | 33,919 | 76 | 5 | 0.04 | 0.08 |
| 146 | HMU03670 | aspA aspartate ammonia-lyase | 51,394 | 94 | 3 | 0.04 | 0.08 |
| 147 | HMU11680 | putative putative MCP-type signal transduction protein | 49,447 | 72 | 3 | 0.04 | 0.07 |
| 148 | HMU04620 | gltA citrate synthase | 51,389 | 53 | 4 | 0.03 | 0.07 |
| 149 | HMU02940 | lon ATP-dependent protease La | 91,485 | 99 | 4 | 0.03 | 0.07 |
| 150 | HMU03400 | ffh signal recognition particle protein | 49,047 | 58 | 2 | 0.03 | 0.07 |
| 151 | HMU06960 | undefined product; COG1426, Uncharacterized protein conserved in bacteria [function unknown]. | 41,075 | 60 | 3 | 0.03 | 0.07 |
| 152 | HMU10220 | undefined product; cd01949: GGDEF diguanylate-cyclase (DGC) or GGDEF domain: | 41,077 | 55 | 3 | 0.03 | 0.07 |
| 153 | HMU09260 | putative putative pyridine nucleotide-disulphide oxidoreductase | 45,027 | 49 | 2 | 0.03 | 0.06 |
| 154 | HMU11870 | fliF flagellar M-ring protein | 63,760 | 54 | 2 | 0.02 | 0.05 |
| 155 | HMU03010 | putative TonB-dependent receptor protein | 89,679 | 60 | 3 | 0.03 | 0.05 |
| 156 | HMU03220 | fusA elongation factor G | 76,896 | 54 | 3 | 0.03 | 0.05 |
| 157 | HMU02190 | ftsH membrane bound zinc metallopeptidase | 69,545 | 87 | 4 | 0.02 | 0.04 |
| 158 | HMU13380 | ppc putative putative phosphoenolpyruvate carboxylase | 101,663 | 51 | 2 | 0.02 | 0.04 |
| 159 | HMU04060 | icd isocitrate dehydrogenase | 82,856 | 75 | 2 | 0.02 | 0.04 |
| 160 | HMU10950 | secA preprotein translocase SECA subunit | 94,354 | 38 | 1 | 0.02 | 0.03 |
| 161 | HMU14010 | undefined product; COG1317/ PRK05687: fliH flagellar biosynthesis/type III secretory pathway protein [cell motility and secretion / intracellular trafficking and secretion] | 92,360 | 50 | 2 | 0.02 | 0.03 |

a. Relative abundance ranked by mol%

b. Score for the entire protein derived by MASCOT, and made up of the individual scores given to each peptide sequence

c. Proportion of each protein sequence identified

d. Exponentially modified protein abundance index. See Methods for details and reference.
