# Supplementary material for: Comparative genomics and proteomics of Helicobacter mustelae, an ulcerogenic and carcinogenic gastric pathogen
Source: BMC Genomics. 2010 Mar 10;11:164. doi: 10.1186/1471-2164-11-164 (PMC2846917; doi:10.1186/1471-2164-11-164)
Supplement: Additional file 7 — The cytosolic proteome of H. mustelae determined by LC-MS [file 1471-2164-11-164-S7.DOCX]

Additional file 7. The cytosolic proteome of *H. mustelae* determined by LC-MS

| **Rank^a^** | **Locus** | **Annotation** | **Mw Da** | **MOWSE Score^b^** | **% Coverage^c^** | **emPAI^d^** | **Mol%** |
| --- | --- | --- | --- | --- | --- | --- | --- |
| 1 | HMU14210 | fldA flavodoxin 1 | 18147 | 620 | 56 | 6.74 | 7.35 |
| 2 | HMU05200 | thiJ 4-methyl-5(beta-hydroxyethyl)-thiazole monophosphate synthesis protein | 20365 | 212 | 32 | 3.64 | 3.97 |
| 3 | HMU01210 | tpx probable thiol peroxidase | 17993 | 886 | 62 | 3.44 | 3.75 |
| 4 | HMU12840 | aroQ 3-dehydroquinate dehydratase | 17477 | 161 | 21 | 2.98 | 3.25 |
| 5 | HMU12860 | sodB superoxide dismutase (Fe) | 24576 | 534 | 45 | 2.41 | 2.63 |
| 6 | HMU00320 | undefined product | 8942 | 162 | 54 | 2.16 | 2.36 |
| 7 | HMU05470 | acpP acyl carrier protein | 8469 | 139 | 48 | 2.16 | 2.36 |
| 8 | HMU03120 | tuf elongation factor TU | 43570 | 1695 | 62 | 2.09 | 2.28 |
| 9 | HMU12690 | possible bacterioferritin | 17198 | 562 | 46 | 1.99 | 2.17 |
| 10 | HMU09770 | trxA thioredoxin | 11553 | 361 | 36 | 1.89 | 2.06 |
| 11 | HMU04180 | hypothetical protein Cj1613c | 30321 | 755 | 29 | 1.71 | 1.87 |
| 12 | HMU03320 | ahpC alkyl hydroperoxide reductase | 22088 | 572 | 44 | 1.68 | 1.83 |
| 13 | HMU14370 | cft ferritin | 19056 | 276 | 45 | 1.42 | 1.55 |
| 14 | HMU00950 | rpsA 30S ribosomal protein S1 | 60196 | 208 | 7 | 1.31 | 1.43 |
| 15 | HMU10800 | lpsJ succinyl-CoA:3-ketoacid-coenzyme A transferase subunit B | 23051 | 501 | 38 | 1.15 | 1.26 |
| 16 | HMU04000 | groEL 60 kD chaperonin (cpn60) | 57443 | 1474 | 48 | 1.02 | 1.11 |
| 17 | HMU03150 | rplK 50S ribosomal protein L11 | 12586 | 473 | 48 | 0.91 | 0.99 |
| 18 | HMU01260 | ald alanine dehydrogenase | 39844 | 662 | 32 | 0.82 | 0.9 |
| 19 | HMU07380 | hupB DNA-binding protein HU homolog | 10122 | 352 | 56 | 0.82 | 0.89 |
| 20 | HMU10120 | peb4[1]f2 major antigenic peptide PEB3ll binding factor 2 | 31816 | 750 | 39 | 0.78 | 0.85 |
| 21 | HMU07050 | rplI 50S ribosomal protein L9 | 16251 | 460 | 49 | 0.74 | 0.81 |
| 22 | HMU04730 | oorB OORB subunit of 2-oxoglutarate:acceptor oxidoreductase | 30613 | 423 | 28 | 0.75 | 0.81 |
| 23 | HMU14090 | putative thioredoxin | 11796 | 267 | 39 | 0.73 | 0.8 |
| 24 | HMU04740 | oorA OORA subunit of 2-oxoglutarate:acceptor oxidoreductase | 40773 | 554 | 36 | 0.71 | 0.77 |
| 25 | HMU02170 | cheY chemotaxis regulatory protein | 13894 | 208 | 25 | 0.70 | 0.76 |
| 26 | HMU11060 | putative putative aminotransferase (nifS protein homolog) | 43365 | 424 | 26 | 0.67 | 0.73 |
| 27 | HMU00290 | fbp putative putative fructose-1,6-bisphosphatase | 30550 | 323 | 20 | 0.67 | 0.73 |
| 28 | HMU03180 | rplL 50S ribosomal protein L7 /L12 | 12946 | 259 | 44 | 0.67 | 0.73 |
| 29 | HMU00100 | putative putative acyl-CoA thioester hydrolase | 16960 | 168 | 20 | 0.64 | 0.7 |
| 30 | HMU13330 | undefined product | 8555 | 166 | 49 | 0.64 | 0.7 |
| 31 | HMU03850 | flaG possible flagellar protein | 14814 | 452 | 34 | 0.63 | 0.69 |
| 32 | HMU03060 | ureB urease alpha subunit | 61146 | 743 | 26 | 0.62 | 0.68 |
| 33 | HMU09760 | trxB thioredoxin reductase | 33972 | 587 | 35 | 0.62 | 0.68 |
| 34 | HMU07390 | ndk nucleoside diphosphate kinase | 15293 | 291 | 25 | 0.62 | 0.67 |
| 35 | HMU05040 | putative hydantoin utilization protein B | 83272 | 948 | 28 | 0.60 | 0.66 |
| 36 | HMU10790 | scoA succinyl-CoA:3-ketoacid-coenzyme A transferase subunit A | 25076 | 305 | 32 | 0.58 | 0.64 |
| 37 | HMU03990 | groES 10 kD chaperonin (cpn10) | 10185 | 129 | 34 | 0.58 | 0.64 |
| 38 | HMU13450 | hypothetical protein Cj0706 | 27047 | 694 | 49 | 0.56 | 0.61 |
| 39 | HMU10960 | katA catalase | 52490 | 781 | 39 | 0.54 | 0.59 |
| 40 | HMU03670 | aspA aspartate ammonia-lyase | 51394 | 620 | 31 | 0.52 | 0.57 |
| 41 | HMU10290 | rpoA DNA-directed RNA polymerase alpha chain | 37551 | 308 | 25 | 0.51 | 0.55 |
| 42 | HMU11440 | putative putative nucleotide phosphoribosyltransferase | 17378 | 164 | 25 | 0.50 | 0.55 |
| 43 | HMU10630 | putative putative periplasmic protein | 21100 | 282 | 24 | 0.48 | 0.53 |
| 44 | HMU05030 | putative putative hydantoinase A | 78444 | 681 | 24 | 0.48 | 0.52 |
| 45 | HMU11200 | glyA serine hydroxymethyltransferase | 45885 | 461 | 24 | 0.48 | 0.52 |
| 46 | HMU08860 | hypB hydrogenase isoenzymes formation protein | 26838 | 328 | 28 | 0.47 | 0.51 |
| 47 | HMU01430 | putative putative periplasmic cytochrome C | 10944 | 178 | 36 | 0.47 | 0.51 |
| 48 | HMU06880 | yabJ putative putative regulatory protein | 13506 | 163 | 26 | 0.47 | 0.51 |
| 49 | HMU09820 | surE SurE protein homolog | 28918 | 352 | 25 | 0.45 | 0.49 |
| 50 | HMU12070 | putative putative exported protein | 16551 | 270 | 33 | 0.45 | 0.49 |
| 51 | HMU14280 | fabD malonyl CoA-acyl carrier protein transacylase | 33947 | 224 | 16 | 0.45 | 0.49 |
| 52 | HMU06890 | rplU 50S ribosomal protein L21 | 11677 | 207 | 29 | 0.45 | 0.49 |
| 53 | HMU05270 | rpsO 30S ribosomal protein S15 | 10411 | 350 | 39 | 0.43 | 0.47 |
| 54 | HMU10410 | rpsH 30S ribosomal protein S8 | 14807 | 305 | 32 | 0.43 | 0.47 |
| 55 | HMU08150 | type I restriction-modification system M protein | 53756 | 521 | 27 | 0.43 | 0.46 |
| 56 | HMU07490 | aspB aspartate aminotransferase | 43332 | 392 | 22 | 0.40 | 0.44 |
| 57 | HMU10190 | putative putative catalase | 36012 | 417 | 17 | 0.40 | 0.43 |
| 58 | HMU03160 | rplA 50S ribosomal protein L1 | 25256 | 390 | 31 | 0.39 | 0.42 |
| 59 | HMU01620 | kdsA 2-dehydro-3-deoxyphosphooctonate aldolase | 29464 | 308 | 27 | 0.39 | 0.42 |
| 60 | HMU07270 | gatC putative putative Glu-tRNAGln amidotransferase subunit C | 10639 | 132 | 25 | 0.39 | 0.42 |
| 61 | HMU01070 | hypothetical protein Cj0449c | 8772 | 91 | 29 | 0.39 | 0.42 |
| 62 | HMU04060 | icd isocitrate dehydrogenase | 82856 | 774 | 28 | 0.37 | 0.41 |
| 63 | HMU03460 | gdh NADP-specific glutamate dehydrogenase | 49935 | 491 | 24 | 0.36 | 0.4 |
| 64 | HMU03030 | undefined product | 35944 | 385 | 28 | 0.36 | 0.4 |
| 65 | HMU06340 | oxidoreductase | 34784 | 433 | 25 | 0.36 | 0.39 |
| 66 | HMU03050 | ureA fusion of urease beta and gamma subunits | 25199 | 340 | 26 | 0.35 | 0.39 |
| 67 | HMU00920 | HIT-family protein | 12412 | 116 | 18 | 0.36 | 0.39 |
| 68 | HMU10340 | infA translation initiation factor IF-1 | 8241 | 116 | 30 | 0.36 | 0.39 |
| 69 | HMU10780 | atoB2 acetyl-CoA acetyltransferase 2 | 44538 | 896 | 47 | 0.35 | 0.38 |
| 70 | HMU10320 | rpsM 30S ribosomal protein S13 | 13576 | 209 | 30 | 0.35 | 0.38 |
| 71 | HMU05620 | ppi peptidyl-prolyl cis-trans isomerase | 18004 | 204 | 31 | 0.35 | 0.38 |
| 72 | HMU05020 | undefined product | 14279 | 171 | 29 | 0.35 | 0.38 |
| 73 | HMU03220 | fusA elongation factor G | 76896 | 565 | 23 | 0.34 | 0.37 |
| 74 | HMU05010 | putative hydantoin hydantoinase A | 70769 | 679 | 23 | 0.33 | 0.36 |
| 75 | HMU11790 | ilvC ketol-acid reductoisomerase | 36381 | 280 | 5 | 0.33 | 0.36 |
| 76 | HMU10470 | undefined product | 5941 | 183 | 26 | 0.33 | 0.36 |
| 77 | HMU02340 | accB putative putative biotin carboxyl carrier protein of acetyl-CoA carboxylase | 16525 | 150 | 18 | 0.33 | 0.36 |
| 78 | HMU02090 | putative putative periplasmic protein | 20737 | 297 | 32 | 0.32 | 0.34 |
| 79 | HMU05990 | putative putative MCP-type signal transduction protein | 47627 | 551 | 23 | 0.30 | 0.33 |
| 80 | HMU10540 | rplD 50S ribosomal protein L4 | 22908 | 287 | 31 | 0.30 | 0.33 |
| 81 | HMU10390 | rplR 50S ribosomal protein L18 | 13407 | 193 | 22 | 0.30 | 0.33 |
| 82 | HMU11070 | nifU protein homolog | 35917 | 327 | 15 | 0.29 | 0.32 |
| 83 | HMU00330 | undefined product | 31033 | 221 | 20 | 0.29 | 0.32 |
| 84 | HMU10400 | rplF 50S ribosomal protein L6 | 19675 | 198 | 27 | 0.29 | 0.32 |
| 85 | HMU10460 | rpsQ 30S ribosomal protein S17 | 9799 | 139 | 24 | 0.29 | 0.32 |
| 86 | HMU04750 | oorD OORD subunit of 2-oxoglutarate:acceptor oxidoreductase | 11559 | 90 | 27 | 0.29 | 0.32 |
| 87 | HMU01180 | putative putative membrane-anchored cell surface protein | 281808 | 943 | 8 | 0.28 | 0.31 |
| 88 | HMU05390 | rpsI 30S ribosomal protein S9 | 14313 | 197 | 28 | 0.28 | 0.3 |
| 89 | HMU06080 | hypothetical protein Cj0372 | 44843 | 128 | 8 | 0.27 | 0.3 |
| 90 | HMU00020 | dnaN DNA polymerase III, beta chain | 41139 | 263 | 20 | 0.25 | 0.28 |
| 91 | HMU10370 | rplO 50S ribosomal protein L15 | 14566 | 260 | 27 | 0.26 | 0.28 |
| 92 | HMU05420 | putative putative pyruvate-flavodoxin oxidoreductase | 44431 | 214 | 14 | 0.26 | 0.28 |
| 93 | HMU00130 | pdxJ putative putative pyridoxal phosphate biosynthetic protein | 30790 | 197 | 10 | 0.26 | 0.28 |
| 94 | HMU02230 | putative putative heavy-metal-associated protein | 7610 | 62 | 19 | 0.26 | 0.28 |
| 95 | HMU04720 | oorC OORC subunit of 2-oxoglutarate:acceptor oxidoreductase | 20113 | 157 | 21 | 0.25 | 0.27 |
| 96 | HMU10510 | rpsS 30S ribosomal protein S19 | 10600 | 128 | 12 | 0.25 | 0.27 |
| 97 | HMU10180 | leuA 2-isopropylmalate synthase | 56022 | 360 | 15 | 0.24 | 0.26 |
| 98 | HMU04620 | gltA citrate synthase | 51389 | 343 | 17 | 0.24 | 0.26 |
| 99 | HMU04350 | atpD ATP synthase F1 sector beta subunit | 51310 | 273 | 13 | 0.24 | 0.26 |
| 100 | HMU01560 | aminotransferase class-V | 41699 | 226 | 15 | 0.23 | 0.25 |
| 101 | HMU03350 | rplS 50S ribosomal protein L19 | 13546 | 193 | 23 | 0.23 | 0.25 |
| 102 | HMU01580 | panC putative putative pantoate--beta-alanine ligase | 30838 | 171 | 18 | 0.23 | 0.25 |
| 103 | HMU01880 | gpsA glycerol-3-phosphate dehydrogenase [NAD(P)+] | 32837 | 164 | 17 | 0.23 | 0.25 |
| 104 | HMU12530 | ssb single-strand DNA binding protein | 18508 | 97 | 19 | 0.23 | 0.25 |
| 105 | HMU10100 | efp elongation factor P | 20986 | 92 | 9 | 0.23 | 0.25 |
| 106 | HMU04690 | tsf elongation factor TS | 39772 | 292 | 17 | 0.22 | 0.24 |
| 107 | HMU01190 | putative hypothetical glycine-rich autotransporter protein | 194616 | 634 | 7 | 0.21 | 0.23 |
| 108 | HMU10550 | rplC 50S ribosomal protein L3 | 21082 | 315 | 27 | 0.21 | 0.23 |
| 109 | HMU06810 | mog molybdopterin biosynthesis protein | 19286 | 59 | 7 | 0.21 | 0.23 |
| 110 | HMU07520 | gatA Glu-tRNAGln amidotransferase subunit A | 49510 | 289 | 17 | 0.20 | 0.22 |
| 111 | HMU07370 | gapA glyceraldehyde 3-phosphate dehydrogenase | 35561 | 225 | 15 | 0.21 | 0.22 |
| 112 | HMU09250 | hypothetical protein Cj1172c | 26698 | 153 | 19 | 0.21 | 0.22 |
| 113 | HMU13360 | tpiA putative putative triosephosphate isomerase | 26212 | 123 | 13 | 0.20 | 0.22 |
| 114 | HMU02260 | putative putative thiamine pyrophosphate enzyme | 61033 | 259 | 13 | 0.20 | 0.21 |
| 115 | HMU07020 | mrp putative putative ATP/GTP-binding protein (mrp protein homolog) | 40316 | 177 | 14 | 0.18 | 0.2 |
| 116 | HMU10530 | rplW 50S ribosomal protein L23 | 10531 | 142 | 24 | 0.19 | 0.2 |
| 117 | HMU10560 | rpsJ 30S ribosomal protein S10 | 11807 | 84 | 23 | 0.19 | 0.2 |
| 118 | HMU04980 | glk glucokinase | 36324 | 69 | 8 | 0.19 | 0.2 |
| 119 | HMU03190 | putative DNA-directed RNA polymerase beta' chain | 323245 | 2001 | 14 | 0.17 | 0.19 |
| 120 | HMU10300 | rpsD 30S ribosomal protein S4 | 24016 | 205 | 28 | 0.18 | 0.19 |
|  | HMU14080 | hypothetical protein Cj1132c | 32714 | 139 | 11 | 0.18 | 0.19 |
| 121 | HMU12540 | rpsF putative putative 30S ribosomal protein S6 | 16404 | 94 | 15 | 0.18 | 0.19 |
| 122 | HMU10720 | putative putative isomerase | 7893 | 90 | 16 | 0.18 | 0.19 |
| 123 | HMU12670 | undefined product | 10754 | 79 | 11 | 0.18 | 0.19 |
| 124 | HMU02100 | gltX2 glutamyl-tRNA synthetase | 52462 | 213 | 15 | 0.17 | 0.18 |
| 125 | HMU05430 | putative pyruvate-flavodoxin oxidoreductase | 35085 | 155 | 12 | 0.17 | 0.18 |
| 126 | HMU01550 | D-3-phosphoglycerate dehydrogenase | 56881 | 125 | 8 | 0.17 | 0.18 |
| 127 | HMU10270 | putative putative NAD(P)H oxidoreductase | 18616 | 79 | 7 | 0.17 | 0.18 |
| 128 | HMU09540 | csrA carbon storage regulator homolog | 8394 | 49 | 17 | 0.17 | 0.18 |
| 129 | HMU06220 | adk adenylate kinase | 21145 | 125 | 10 | 0.16 | 0.17 |
| 130 | HMU02060 | undefined product | 32627 | 114 | 10 | 0.16 | 0.17 |
| 131 | HMU01840 | undefined product | 23353 | 61 | 13 | 0.16 | 0.17 |
| 132 | HMU02960 | hypothetical protein Cj1075 | 17585 | 48 | 7 | 0.15 | 0.17 |
| 133 | HMU09900 | hydB Ni/Fe-hydrogenase large subunit | 64429 | 217 | 13 | 0.15 | 0.16 |
| 134 | HMU01530 | undefined product | 23926 | 195 | 14 | 0.15 | 0.16 |
| 135 | HMU12170 | guaA GMP synthase (glutamine-hydrolyzing) | 58032 | 163 | 8 | 0.15 | 0.16 |
| 136 | HMU10450 | rplN 50S ribosomal protein L14 | 13373 | 143 | 20 | 0.15 | 0.16 |
| 137 | HMU12520 | rpsR 30S ribosomal protein S18 | 10360 | 114 | 26 | 0.15 | 0.16 |
| 138 | HMU10110 | putative putative fructose-1,6-bisphosphate aldolase | 33782 | 104 | 11 | 0.15 | 0.16 |
| 139 | HMU02430 | hypothetical protein Cj0459c | 9393 | 55 | 10 | 0.15 | 0.16 |
| 140 | HMU11760 | ilvD putative putative dihydroxy-acid dehydratase | 59872 | 195 | 9 | 0.14 | 0.15 |
| 141 | HMU02890 | zinc protease-like protein | 49034 | 179 | 11 | 0.13 | 0.15 |
| 142 | HMU13960 | putative putative phospho-sugar mutase | 51097 | 144 | 9 | 0.14 | 0.15 |
| 143 | HMU06210 | ppa inorganic pyrophosphatase | 19702 | 116 | 14 | 0.14 | 0.15 |
| 144 | HMU13040 | ilvE branched-chain amino acid aminotransferase | 34251 | 89 | 14 | 0.14 | 0.15 |
| 145 | HMU08240 | undefined product | 12498 | 66 | 9 | 0.14 | 0.15 |
| 146 | HMU14430 | ileS isoleucyl-tRNA synthetase | 105368 | 326 | 10 | 0.13 | 0.14 |
| 147 | HMU11980 | fabG 3-oxoacyl-[acyl-carrier protein] reductase | 26634 | 184 | 18 | 0.13 | 0.14 |
| 148 | HMU10170 | leuB 3-isopropylmalate dehydrogenase | 38714 | 174 | 10 | 0.13 | 0.14 |
| 149 | HMU05050 | undefined product | 30228 | 158 | 12 | 0.13 | 0.14 |
| 150 | HMU05910 | pyrH uridylate kinase | 26248 | 112 | 12 | 0.13 | 0.14 |
| 151 | HMU03500 | putative putative amino-acid transporter periplasmic solute-binding protein | 30533 | 89 | 14 | 0.13 | 0.14 |
| 152 | HMU01860 | eno enolase | 46416 | 72 | 9 | 0.13 | 0.14 |
| 153 | HMU00440 | fabZ (3R)-hydroxymyristoyl-[acyl carrier protein] dehydratase | 14472 | 51 | 8 | 0.13 | 0.14 |
| 154 | HMU04800 | undefined product | 16005 | 51 | 9 | 0.13 | 0.14 |
| 155 | HMU12490 | conserved hypothetical protein | 23846 | 37 | 9 | 0.13 | 0.14 |
| 156 | HMU02150 | putA putative putative proline dehydrogenase/delta-1-pyrroline-5-carboxylate dehydrogenase | 133560 | 298 | 8 | 0.12 | 0.13 |
| 157 | HMU02280 | argS arginyl-tRNA synthetase | 61644 | 208 | 11 | 0.12 | 0.13 |
| 158 | HMU04660 | hypothetical protein Cj0172c | 45067 | 145 | 9 | 0.12 | 0.13 |
| 159 | HMU10350 | map methionine aminopeptidase | 27492 | 74 | 8 | 0.12 | 0.13 |
| 160 | HMU01630 | ribH 6,7-dimethyl-8-ribityllumazine synthase | 17353 | 72 | 9 | 0.12 | 0.13 |
| 161 | HMU08090 | hypothetical protein Cj0667 | 9356 | 53 | 11 | 0.12 | 0.13 |
| 162 | HMU09620 | frr ribosome recycling factor | 21230 | 138 | 15 | 0.11 | 0.12 |
| 163 | HMU00760 | undefined product | 27997 | 130 | 13 | 0.11 | 0.12 |
| 164 | HMU09190 | undefined product | 13197 | 88 | 12 | 0.11 | 0.12 |
| 165 | HMU08470 | hypothteical protein Cj1100 | 16482 | 40 | 8 | 0.11 | 0.12 |
| 166 | HMU12630 | proS prolyl-tRNA synthetase | 65263 | 303 | 12 | 0.11 | 0.11 |
| 167 | HMU06520 | pepA aminopeptidase | 52814 | 205 | 11 | 0.10 | 0.11 |
| 168 | HMU02050 | purA adenylosuccinate synthetase | 46238 | 179 | 9 | 0.10 | 0.11 |
| 169 | HMU07940 | putative putative D-2-hydroxyacid dehydrogenase | 35020 | 150 | 11 | 0.10 | 0.11 |
| 170 | HMU04950 | edd phosphogluconate dehydratase | 65771 | 148 | 6 | 0.11 | 0.11 |
| 171 | HMU13180 | gmd GDP-mannose 4,6-dehydratase | 45784 | 128 | 10 | 0.10 | 0.11 |
| 172 | HMU07640 | undefined product | 38765 | 76 | 9 | 0.11 | 0.11 |
| 173 | HMU10380 | rpsE 30S ribosomal protein S5 | 15592 | 62 | 8 | 0.10 | 0.11 |
| 174 | HMU09830 | ispA geranyltranstransferase | 32847 | 56 | 4 | 0.10 | 0.11 |
| 175 | HMU04410 | pal peptidoglycan associated lipoprotein (omp18) | 18949 | 47 | 8 | 0.10 | 0.11 |
| 176 | HMU11920 | nikR nickel responsive regulator | 17179 | 46 | 6 | 0.10 | 0.11 |
| 177 | HMU06570 | dnaK heat shock protein dnaK | 67854 | 412 | 13 | 0.09 | 0.1 |
| 178 | HMU13690 | ABC transporter ATP-binding protein | 61452 | 210 | 8 | 0.09 | 0.1 |
| 179 | HMU07510 | guaB inosine-5'-monophosphate dehydrogenase | 52019 | 147 | 8 | 0.10 | 0.1 |
| 180 | HMU05840 | putative flagellin | 53982 | 133 | 7 | 0.09 | 0.1 |
| 181 | HMU14040 | putative putative zinc protease | 49968 | 123 | 9 | 0.09 | 0.1 |
| 182 | HMU01400 | putative putative phospho-sugar mutase | 49236 | 89 | 8 | 0.09 | 0.1 |
| 183 | HMU01920 | htrA serine protease (protease DO) | 45914 | 60 | 7 | 0.09 | 0.1 |
| 184 | HMU06580 | grpE heat shock protein grpE | 23377 | 42 | 6 | 0.09 | 0.1 |
| 185 | HMU04970 | pgl 6-phosphogluconolactonase | 25069 | 40 | 5 | 0.09 | 0.1 |
| 186 | HMU00630 | putative putative autotransporter protein | 151727 | 155 | 3 | 0.08 | 0.09 |
| 187 | HMU03640 | galU UTP--glucose-1-phosphate uridylyltransferase | 30646 | 85 | 4 | 0.08 | 0.09 |
| 188 | HMU01090 | fumA fumarate hydratase, subunit A | 30210 | 82 | 7 | 0.08 | 0.09 |
| 189 | HMU13850 | hypothetical protein Cj0026c | 23845 | 64 | 9 | 0.08 | 0.09 |
| 190 | HMU04940 | eda 7 KHG/KDPG aldolase | 22437 | 57 | 6 | 0.08 | 0.09 |
| 191 | HMU06970 | putative putative periplasmic protein | 31947 | 55 | 5 | 0.08 | 0.09 |
| 192 | HMU02180 | prmA possible ribosomal protein methyltransferase | 33607 | 46 | 4 | 0.09 | 0.09 |
| 193 | HMU08080 | ctc protein homolog | 19574 | 37 | 5 | 0.08 | 0.09 |
| 194 | HMU13630 | undefined product | 32670 | 34 | 4 | 0.08 | 0.09 |
| 195 | HMU06450 | (folD methylenetetrahydrofolate dehydrogenase/methenyltetrahydrofolate cyclohydrolase | 30354 | 33 | 10 | 0.08 | 0.09 |
| 196 | HMU13820 | argG argininosuccinate synthase | 47531 | 181 | 8 | 0.07 | 0.08 |
| 197 | HMU04860 | putative alanine dehydrogenase 1 | 41219 | 117 | 7 | 0.07 | 0.08 |
| 198 | HMU14410 | glmS glucosamine--fructose-6-phosphate aminotransferase (isomerizing) | 66162 | 105 | 5 | 0.07 | 0.08 |
| 199 | HMU09630 | pyrE putative putative orotate phosphoribosyltransferase | 23152 | 73 | 9 | 0.07 | 0.08 |
| 200 | HMU14020 | putative putative oxidoreductase | 28557 | 72 | 6 | 0.07 | 0.08 |
| 201 | HMU13940 | putative putative exported protein | 29374 | 61 | 7 | 0.07 | 0.08 |
| 202 | HMU04560 | undefined product | 30374 | 60 | 6 | 0.07 | 0.08 |
| 203 | HMU02070 | prsA ribose-phosphate pyrophosphokinase | 33819 | 58 | 5 | 0.07 | 0.08 |
| 204 | HMU07770 | nitroreductase family protein | 24182 | 55 | 7 | 0.07 | 0.08 |
| 205 | HMU03170 | rplJ 50S ribosomal protein L10 | 18003 | 53 | 6 | 0.07 | 0.08 |
| 206 | HMU12900 | modA putative putative molybdate-binding lipoprotein | 27357 | 35 | 6 | 0.07 | 0.08 |
| 207 | HMU09460 | hypothetical protein Cj0957c | 26037 | 34 | 6 | 0.07 | 0.08 |
| 208 | HMU11190 | lysS lysyl-tRNA synthetase | 58552 | 119 | 6 | 0.06 | 0.07 |
| 209 | HMU05380 | rplM 50S ribosomal protein L13 | 15981 | 77 | 12 | 0.06 | 0.07 |
| 210 | HMU05480 | fabF 3-oxoacyl-[acyl-carrier-protein] synthase | 43136 | 56 | 5 | 0.07 | 0.07 |
| 211 | HMU07860 | rplT 50S ribosomal protein L20 | 14462 | 49 | 7 | 0.06 | 0.07 |
| 212 | HMU00090 | galE UDP-glucose 4-epimerase | 37991 | 47 | 3 | 0.06 | 0.07 |
| 213 | HMU11800 | ilvA threonine dehydratase biosynthetic | 45863 | 37 | 4 | 0.06 | 0.07 |
| 214 | HMU00340 | phosphate acetyl/butaryl transferase | 32120 | 34 | 8 | 0.07 | 0.07 |
| 215 | HMU05550 | acnB aconitate hydratase | 92749 | 124 | 5 | 0.05 | 0.06 |
| 216 | HMU07780 | typA GTP-binding protein typA homolog | 72240 | 120 | 6 | 0.05 | 0.06 |
| 217 | HMU10240 | tig trigger factor (peptidyl-prolyl cis /trans isomerase, chaperone) | 53071 | 98 | 5 | 0.05 | 0.06 |
| 218 | HMU09200 | putative putative two-component regulator | 26029 | 79 | 7 | 0.06 | 0.06 |
| 219 | HMU07190 | periplasmic solute binding protein for ABC transport system | 33098 | 73 | 5 | 0.05 | 0.06 |
| 220 | HMU10880 | tctC tricarboxylic transport protein | 35223 | 67 | 4 | 0.06 | 0.06 |
| 221 | HMU08980 | undefined product | 26863 | 60 | 7 | 0.06 | 0.06 |
| 222 | HMU09380 | purC phosphoribosylaminoimidazole-succinocarboxamide synthase | 27288 | 57 | 6 | 0.06 | 0.06 |
| 223 | HMU14230 | glnA glutamine synthetase | 53872 | 51 | 4 | 0.06 | 0.06 |
| 224 | HMU02660 | putative putative molybdopterin-binding oxidoreductase | 37967 | 38 | 6 | 0.06 | 0.06 |
| 225 | HMU07310 | possible restriction /modification enzyme | 150662 | 262 | 4 | 0.05 | 0.05 |
| 226 | HMU10050 | leuS leucyl-tRNA synthetase | 92515 | 145 | 4 | 0.05 | 0.05 |
| 227 | HMU05180 | htpG hsp90 family heat shock protein | 71179 | 127 | 6 | 0.04 | 0.05 |
| 228 | HMU07970 | ppsA putative putative phosphoenolpyruvate synthase | 92171 | 124 | 5 | 0.04 | 0.05 |
| 229 | HMU03770 | cheA chemotaxis histidine kinase | 85293 | 89 | 5 | 0.05 | 0.05 |
| 230 | HMU09370 | putative putative secreted protease | 54758 | 85 | 7 | 0.05 | 0.05 |
| 231 | HMU05700 | tkt transketolase | 70008 | 75 | 3 | 0.05 | 0.05 |
| 232 | HMU07360 | pgk phosphoglycerate kinase | 44544 | 64 | 4 | 0.05 | 0.05 |
| 233 | HMU10490 | rpsC 30S ribosomal protein S3 | 25408 | 63 | 8 | 0.04 | 0.05 |
| 234 | HMU07600 | pgm phosphoglycerate mutase | 54862 | 62 | 4 | 0.05 | 0.05 |
| 235 | HMU12610 | hemC porphobilinogen deaminase | 34693 | 60 | 5 | 0.05 | 0.05 |
| 236 | HMU01870 | recA recA protein | 37765 | 57 | 5 | 0.05 | 0.05 |
| 237 | HMU10520 | rplB 50S ribosomal protein L2 | 29869 | 55 | 4 | 0.05 | 0.05 |
| 238 | HMU12850 | argM succinylornithine transaminase | 45031 | 43 | 6 | 0.04 | 0.05 |
| 239 | HMU12100 | undefined product | 69100 | 37 | 2 | 0.04 | 0.05 |
| 240 | HMU01770 | infB translation initiation factor IF-2 | 95900 | 140 | 4 | 0.03 | 0.04 |
| 241 | HMU11990 | putative putative type IIS restriction /modification enzyme, N-terminal half | 203714 | 122 | 2 | 0.04 | 0.04 |
| 242 | HMU11000 | molybdopterin-containing oxidoreductase | 90256 | 116 | 4 | 0.04 | 0.04 |
| 243 | HMU02120 | speA biosynthetic arginine decarboxylase | 70190 | 92 | 3 | 0.04 | 0.04 |
| 244 | HMU02900 | gatB Glu-tRNAGln amidotransferase subunit B | 52808 | 89 | 3 | 0.03 | 0.04 |
| 245 | HMU08630 | putative putative outer membrane autotransporter | 155228 | 75 | 1 | 0.04 | 0.04 |
| 246 | HMU01970 | pyrG CTP synthase | 60271 | 70 | 4 | 0.04 | 0.04 |
| 247 | HMU07550 | putative putative surface-anchored protein | 64957 | 60 | 2 | 0.04 | 0.04 |
| 248 | HMU02350 | accC biotin carboxylase | 50467 | 55 | 4 | 0.04 | 0.04 |
| 249 | HMU08410 | argH argininosuccinate lyase | 52072 | 51 | 3 | 0.04 | 0.04 |
| 250 | HMU04100 | trpD anthranilate synthase component II; anthranilate phosphoribosyltransferase | 59587 | 48 | 4 | 0.04 | 0.04 |
| 251 | HMU11680 | putative putative MCP-type signal transduction protein | 49447 | 48 | 4 | 0.04 | 0.04 |
| 252 | HMU02720 | serS seryl-tRNA synthetase | 49005 | 47 | 3 | 0.04 | 0.04 |
| 253 | HMU08510 | rho transcription termination factor | 48946 | 46 | 5 | 0.03 | 0.04 |
| 254 | HMU04990 | pgi1 glucose-6-phosphate isomerase | 62475 | 35 | 2 | 0.04 | 0.04 |
| 255 | HMU04330 | atpA ATP synthase F1 sector alpha subunit | 55012 | 34 | 2 | 0.03 | 0.04 |
| 256 | HMU10220 | undefined product | 41077 | 33 | 33 | 0.04 | 0.04 |
| 257 | HMU04070 | putative type I restriction-modification system S protein | 50085 | 90 | 5 | 0.03 | 0.03 |
| 258 | HMU06230 | aspS aspartyl-tRNA synthetase | 65739 | 54 | 3 | 0.03 | 0.03 |
| 259 | HMU07470 | pnp polyribonucleotide nucleotidyltransferase | 76761 | 52 | 2 | 0.03 | 0.03 |
| 260 | HMU14140 | metS methionyl-tRNA synthetase | 72821 | 43 | 3 | 0.03 | 0.03 |
| 261 | HMU07140 | topA DNA topoisomerase I | 82811 | 70 | 1 | 0.02 | 0.02 |
| 262 | HMU01940 | valS valyl-tRNA synthetase | 101625 | 47 | 2 | 0.02 | 0.02 |
| 263 | HMU10950 | secA preprotein translocase SECA subunit | 94354 | 46 | 2 | 0.02 | 0.02 |
| 264 | HMU02940 | lon ATP-dependent protease La | 91485 | 38 | 2 | 0.02 | 0.02 |
| 265 | HMU08170 | type I restriction enzyme | 117545 | 46 | 5 | 0.01 | 0.01 |

a. Relative abundance ranked by mol%

b. Score for the entire protein derived by MASCOT, and made up of the individual scores given to each peptide sequence

c. Proportion of each protein sequence identified

d. Exponentially modified protein abundance index. See Methods for details and reference.
