# Supplementary material for: Comparative genomics and proteomics of Helicobacter mustelae, an ulcerogenic and carcinogenic gastric pathogen
Source: BMC Genomics. 2010 Mar 10;11:164. doi: 10.1186/1471-2164-11-164 (PMC2846917; doi:10.1186/1471-2164-11-164)
Supplement: Additional file 8 — Motifs associated with highly expressed genes in the H. mustelae cell envelope proteome [file 1471-2164-11-164-S8.DOCX]

Additional file 8. Motifs associated with highly expressed genes in the *H. mustelae* cell envelope proteome
