# Supplementary material for: Comparative genomics and proteomics of Helicobacter mustelae, an ulcerogenic and carcinogenic gastric pathogen
Source: BMC Genomics. 2010 Mar 10;11:164. doi: 10.1186/1471-2164-11-164 (PMC2846917; doi:10.1186/1471-2164-11-164)
Supplement: Additional file 9 — Motifs associated with highly expressed genes in the H. mustelae cytosol proteome [file 1471-2164-11-164-S9.DOCX]

Additional file 9. Motifs associated with highly expressed genes in the *H. mustelae* cytosol proteome
