# Supplementary material for: Comparative genomics and proteomics of Helicobacter mustelae, an ulcerogenic and carcinogenic gastric pathogen
Source: BMC Genomics. 2010 Mar 10;11:164. doi: 10.1186/1471-2164-11-164 (PMC2846917; doi:10.1186/1471-2164-11-164)
Supplement: Additional file 10 — Orthologue comparisons between selected Campylobacterales, and T. maritima as out-group. The lower triangle indicates the total number of orthologs in genome-genome comparisons while the upper triangle shows the average sequence identity values expressed in percentages. [file 1471-2164-11-164-S10.DOCX]

Additional file 10. Orthologue comparisons between selected *Campylobacterales*, and *T. maritima* as out-group. The lower triangle indicates the total number of orthologs in genome-genome comparisons while the upper triangle shows the average sequence identity values expressed in percentages.

|  | *H. mustelae* | *H. pylori* | *H. hepaticus* | *C. coli* | *C. jejuni* | *C. lari* | *C. uppsaliensis* | *W. succinogenes* | *T. maritima* |
| --- | --- | --- | --- | --- | --- | --- | --- | --- | --- |
| *H. mustelae* | X | 56.87 | 57.06 | 51.49 | 51.53 | 51.37 | 51.78 | 56.01 | 41.75 |
| *H. pylori* | 760 | X | 55.97 | 51.04 | 51.29 | 51.09 | 51.09 | 54.91 | 42.16 |
| *H. hepaticus* | 828 | 788 | X | 51.95 | 51.92 | 51.91 | 52.12 | 57.01 | 42.1 |
| *C. coli* | 684 | 653 | 785 | X | 85.98 | 69.43 | 74.26 | 52.6 | 42.56 |
| *C. jejuni* | 685 | 648 | 785 | 1321 | X | 69.41 | 75.12 | 52.68 | 42.68 |
| *C. lari* | 672 | 660 | 748 | 1105 | 1139 | X | 67.49 | 52.5 | 42.21 |
| *C. uppsaliensis* | 652 | 648 | 741 | 1191 | 1181 | 1074 | X | 52.36 | 42.24 |
| *W. succinogenes* | 736 | 716 | 860 | 776 | 794 | 767 | 760 | X | 42.39 |
| *T. maritima* | 251 | 222 | 256 | 259 | 262 | 252 | 253 | 292 | X |
