# Supplementary material for: Comparative genomics and proteomics of Helicobacter mustelae, an ulcerogenic and carcinogenic gastric pathogen
Source: BMC Genomics. 2010 Mar 10;11:164. doi: 10.1186/1471-2164-11-164 (PMC2846917; doi:10.1186/1471-2164-11-164)
Supplement: Additional file 11 — Presence in H. mustelae of orthologues of H. pylori genes identified as essential for colonization of the Mongolian gerbil. [file 1471-2164-11-164-S11.DOCX]

Additional file 11. Presence in *H. mustelae* of orthologues of *H. pylori* genes identified as essential for colonization of the Mongolian gerbil.

| ***H. pylori* gene no.** | **Gene name** | **Predicted function** | ***H. mustelae* ortholog, % identity (BLAST alignment)** |
| --- | --- | --- | --- |
| **Motility and chemotaxis** | | | |
| 0601a | *flaA* | Flagellin A | HMU 05840 73% |
| 0295 |  | Flagellin B homologue | HMU06870 49% |
| 1558a | *flgC* | Flagellar basal-body rod protein | HMU12440 74% |
| 0907a | *flgD* | Hook assembly protein | HMU07810 65% |
| 0870a | *flgE* | Flagellar hook | HMU00850 71% |
| 1092 | *flgG* | Flagellar basal-body rod protein | HMU01910 78% |
| 0325a | *flgH* | Flagellar L-ring | HMU11740 61% |
| 1119a | *flgK* | Flagellar hook-associated protein 1 | HMU04500 57% |
| 0752a | *fliD* | Flagellar hook-associated protein 2 | HMU03840 51% |
| 0351a | *fliF* | Flagellar basal-body M-ring protein | HMU11870 59% |
| 1420a | *fliI* | Flagellar export ATPase | HMU10250 68% |
| 0685 | *fliP* | Flagellar biosynthetic protein | HMU02400 75% |
| 0753a | *fliS* | Flagellar protein | HMU03830 71% |
| 0797a | *hpaA* | Flagellar sheath adhesin | Not found |
| 0232a |  | Secreted protein | HMU06070 49% |
| 0392a | *cheA* | Histidine kinase | HMU03770 67% |
| 0393a | *cheV* | Chemotaxis protein | HMU06240 24% |
| **Cell envelope and outer membrane proteins** | | | |
| 0360 | *galE* | UDP-glucose4-epimerase | HMU00090 55% |
| 0366 |  | Spore coat polysaccharide biosynthesis protein C | HMU06610 23% |
| 0788 | *omp* | Outer membrane protein | HMU05650 36% |
| 0254a | *omp8* | Outer membrane protein | HMU04930? 20% |
| **Other system or transport systems** | | | |
| 0017 | *comB4* | Natural competence-associated typeIV transport system; virB4 homolog | Not found |
| 1421a | *trbB* | virB11 homolog | Not found |
| 0055a | *putP* | Prolinepermease | HMU02140 64% |
| 0302a | *dppF* | Dipeptide ABC transporter ATP-binding protein | HMU10890 93% |
| 1091a | *kgtP* | Alpha-ketoglutarate permease | HMU13610 52% |
| 1082a | *msbA* | Multidrug resistance protein | HMU02240 56% |
| 1206a | *hetA* | Multidrug resistance protein | HMU00890 45% |
| 1506a | *gltS* | Glutamate permease | HMU08190 65% |
| **Stress response and acid survival** | | | |
| 0073a | *ureA* | Urease alpha subunit | HMU03050 70% |
| 0072a | *ureB* | Urease beta subunit (urea amidohydrolase) | HMU0306 78% |
| 0067a | *ureH* | Urease accessoryprotein | HMU03110 48% |
| 0071a | *ureI* | Urease accessoryprotein, urea transporter | HMU03070 58% |
| **Regulatory functions** | | | |
| 0714 | *rpoN* | RNApolymerasesigma54factor | HMU10620 55% |
| 0930a | *surE* | Stationary phase survival protein | HMU09820 52% |
| **Central intermediary metabolism and amino acid biosynthesis** | | | |
| 0237a | *hemC* | Porphobilinogen deaminase | HMU12610 61% |
| 0397a | *serA* | Phosphoglycerate dehydrogenase | HMU01550 51% |
| **Protein degradation** | | | |
| 0169a | *prtC* | Collagenase | HMU02630 75% |
| **VacA paralogues** | | | |
| 0289a |  | VacA-paralogue | HMU06680 37% |
| **Hypothetical proteins** | | | |
| 0245a |  |  | Not found |
| 0288a |  |  | HMU03800 40% |
| 0350a |  |  | HMU07280 32% |
| 0486 |  |  | HMU0564 30% |
| 0973 |  |  | HMU07610 40% |
| 1525 |  |  | Not found |
| 0758a |  |  | HMU0602 45% |
| 1486 |  |  | HMU13730 34% |
